# Identification of Bioactive Peptides from *Caenorhabditis elegans* Secretions That Promote Indole-3-Acetic Acid Production in *Arthrobacter pascens* ZZ21

**DOI:** 10.3390/microorganisms13081951

**Published:** 2025-08-21

**Authors:** Shan Sun, Mengsha Li, Luchen Tao, Xiran Liu, Lei Ouyang, Gen Li, Feng Hu, Huixin Li

**Affiliations:** 1 The Sanya Institute of Nanjing Agricultural University, Nanjing Agricultural University, Sanya 572025, China; 2019203039@njau.edu.cn (S.S.); fenghu@njau.edu.cn (F.H.); 2 Soil Ecology Laboratory, College of Resources and Environmental Sciences, Nanjing Agricultural University, Nanjing 211800, China; tlc@stu.njau.edu.cn (L.T.); liuxiran@stu.njau.edu.cn (X.L.); 13560692510@163.com (L.O.); t2021113@njau.edu.cn (G.L.); 3 Ningbo Key Laboratory of Agricultural Germplasm Resources Mining and Environmental Regulation, College of Science and Technology, Ningbo University, Cixi 315300, China; 4 Jiangsu Collaborative Innovation Center for Solid Organic Waste Resource Utilization, Nanjing 210095, China

**Keywords:** *Caenorhabditis elegans*, peptides, indole-3-acetic acid, plant growth-promoting bacterium

## Abstract

*Caenorhabditis elegans*, a free-living nematode model, secretes neuropeptides, but the ecological roles of its peptide exudates in regulating rhizosphere microbial activity remain largely unexplored. We identified six short peptides (P1, P9, P19, P20, P25, and P26) from *C. elegans* exudates that significantly enhanced indole-3-acetic acid (IAA) production by the plant growth-promoting bacterium *Arthrobacter pascens* ZZ21. These peptides were heat-labile and proteinase K-sensitive but unaffected by DNase I or RNase A, confirming their proteinaceous (peptide) nature rather than nucleic acid origin. The retention of bioactivity in n-butanol extracts further supported their hydrophilic, peptide-like properties. LC-MS/MS identified 30 linear peptides, including the six bioactive ones, which exhibited distinct dose-dependent effects, suggesting diverse regulatory mechanisms. Despite their relatively low abundance, these peptides strongly promoted IAA production in the bacterial culture system across multiple concentrations. These findings reveal an unrecognized mechanism whereby free-living nematodes regulate rhizobacterial metabolism via secreted peptides, offering new insights into nematode-mediated chemical signaling. Therefore, this study advances understanding of plant–microbe–nematode interactions and highlights strategies for manipulating rhizosphere microbiota in sustainable agriculture.

## 1. Introduction

Nematodes are a vital component of rhizosphere biodiversity, comprising not only harmful plant–parasitic species but also free-living nematodes with essential ecological roles [1,2]. Among global soil ecosystems, bacterivorous free-living nematodes represent the most abundant trophic group, accounting for nearly half of all soil nematodes [3,4]. Their grazing enhances bacterial dispersal and resource redistribution—particularly under uneven nutrient distribution or high microhabitat heterogeneity [5]. They also promote nutrient cycling by mineralizing nitrogen and transferring carbon and nitrogen to higher trophic levels, thereby improving soil turnover and material flow [6,7]. Notably, bacterivorous nematodes regulate rhizosphere microbial communities, stimulate plant growth-promoting bacteria, and indirectly support plant growth—likely through improved nutrient use efficiency and increased phytohormone production such as indole-3-acetic acid (IAA) [3,8,9]. As key ecological regulators, they play a critical role in plant–microbe interaction networks, nutrient recycling, and long-term soil and plant health.

Importantly, nematodes contribute to soil ecology beyond their feeding activities. They secrete diverse small molecules—including metabolic byproducts, ascarosides, and peptide hormones—that act as chemical signals in the soil. These secretions regulate nematode behavior and communication, modulate microbial activity, trigger trap formation in predatory fungi, and even activate plant immune responses, enabling cross-kingdom communication among soil organisms [10,11,12,13,14]. Previous studies have shown that bacterivorous nematodes significantly enhance bacterial activity and increase soil IAA concentrations [9,15,16]. IAA, a naturally occurring auxin produced by a wide range of organisms, including plants, fungi, bacteria, and microalgae, is central to plant growth and development regulation [17,18,19,20,21]. In the rhizosphere, IAA from beneficial bacteria acts as a signaling molecule that promotes plant growth, improves stress tolerance, and strengthens plant–microbe interactions [22,23,24]. Our previous research found that exudates from the free-living nematode *Caenorhabditis elegans* significantly increased IAA production by the plant growth-promoting bacterium *Arthrobacter pascens* ZZ21, primarily by activating the indole-3-pyruvic acid (IPyA) biosynthetic pathway, as indicated by elevated gene expression and IPyA accumulation [25]. These findings suggest that nematode-derived secretions may serve as regulatory signals mediating metabolic interactions between bacterivorous nematodes and beneficial microbes. However, the chemical nature and molecular mechanisms of these bioactive compounds remain largely unknown and require further investigation.

This study aims to identify bioactive compounds in *C. elegans* exudates that promote IAA production in *A. pascens* ZZ21. The physicochemical properties of the active substances were preliminarily assessed through heat stability, enzymatic sensitivity, and solvent extraction assays. LC-MS/MS was then used to identify and analyze the structure of the active components, followed by chemical synthesis of selected candidates for activity validation. This research offers new insights into how nematodes regulate beneficial microbial metabolism and provides a theoretical basis for understanding signal transduction in the plant–microbe–nematode interaction network.

## 2. Materials and Methods

### 2.1. C. elegans Culture and Collection of Excretions

The *C. elegans* wild-type strain N2 (Figure S1) was obtained from the Caenorhabditis Genetics Center (CGC) and maintained at 20 °C on nematode growth medium (NGM) agar plates seeded with *Escherichia coli* OP50. NGM was prepared by combining 3 g NaCl, 2.5 g peptone, 17 g agar, and 975 mL H_2_O, followed by autoclaving. After sterilization, it was supplemented with 25 mL of 1 M KPO_4_ buffer (pH 6.0) and 1 mL each of 1 M CaCl_2_, 1 M MgSO_4_, and 5 mg/mL cholesterol (filter-sterilized through a 0.22 μm membrane) [25]. Once *C. elegans* reached the desired stage, nematodes were transferred to M9 buffer (5 g NaCl, 3 g KH_2_PO_4_, 6 g Na_2_HPO_4_, and 1 mL of 1 M MgSO_4_, adjusted to 1 L and autoclaved) using a modified Baermann funnel method. Surface bacteria were removed by three washes with M9 buffer (1500× *g*, 22 °C, 3 min each) [26,27,28]. Gut bacteria were eliminated by incubating nematodes in M9 buffer at 22 °C with shaking (250 rpm) for 30 min, followed by rinsing with sterile water. The suspension was adjusted to 15,000 individuals/mL in sterile water. Nematode excretions were extracted via ultrasonication using a probe-type sonicator (Model XO-1000D, Nanjing Xianou Instrument Manufacturing Co., Ltd., Nanjing, China; 60% power, 3 s pulse with 3 s intervals for 45 min) [25], centrifuged at 1500× *g* for 5 min, and the supernatant was filtered (0.22 μm membrane), lyophilized, and stored at −80 °C for bioassays.

### 2.2. Bacterial Cultivation and IAA Quantification

The plant growth-promoting strain *Arthrobacter pascens* ZZ21 (China General Microbiology Culture Collection Center, CGMCC accession no. 7325), isolated from forest soil in Zijin Mountain, Nanjing, Jiangsu, China, was preserved in our lab. It is a Gram-positive *Arthrobacter* that forms circular, convex, smooth, opaque, and creamy-white to pale-yellow colonies on nutrient agar. It was cultured in an inorganic salt liquid medium with 200 mg/L L-tryptophan. The medium contained 5 g glucose, 2 g (NH_4_)_2_SO_4_, 0.5 g NaH_2_PO_4_, 0.5 g K_2_HPO_4_, 0.2 g MgSO_4_·7H_2_O, and 0.1 g CaCl_2_·2H_2_O per liter of distilled water, pH 7.0 (Li M S, 2021), and incubated at 30 °C with shaking at 250 rpm.

Following incubation, bacterial cells were removed by centrifugation (8000 rpm, 10 min). The supernatant was mixed with an equal volume of Salkowski reagent (50 mL 35% HClO_4_ + 1 mL 0.5 M FeCl_3_), incubated at room temperature in the dark for 30 min, and absorbance was measured at 530 nm to determine IAA concentration using a standard curve [18,29].

### 2.3. Preliminary Screening of Bioactive Compounds in Nematode Excretions

To investigate the bioactive compounds in *C. elegans* N2 exudates that promote IAA production by *A. pascens* ZZ21, the exudates underwent heat treatment (100 °C, 10 min) and digestion with DNase I, RNase A, and proteinase K. To further characterize the nature of the bioactive compounds, the nematode exudate dissolved in sterile water was individually mixed with each of five organic solvents of varying polarity—n-butanol, ethyl acetate, chloroform, toluene, and n-hexane—at a 1:1 volume ratio. After standing at room temperature for 1 h to allow phase separation, the aqueous phase was subjected to a second extraction with the same solvent and volume. The two resulting organic phases were pooled and dried under a stream of nitrogen. The dried residues were reconstituted in an equal volume of sterile water and filtered through a 0.22 μm membrane for subsequent bioactivity screening. A portion of the n-butanol extract, reconstituted in sterile water, was then subjected to proteinase K treatment to assess whether the active compounds were peptide-like. All treated samples were stored at –80 °C for later analysis. For bioactivity assays, 1% *A. pascens* ZZ21 seed culture was inoculated into 30 mL of inorganic salt medium with 200 mg/L L-tryptophan. The treated exudate samples were added at 2000 WE/mL, where one worm equivalent (WE) refers to the lysate obtained from one individual *C. elegans* [25]. Cultures were incubated at 30 °C, 250 rpm for 48 h, after which IAA concentration in the supernatant was measured.

### 2.4. Peptide Identification

To identify bioactive peptides responsible for the growth-promoting activity, the most effective bioassay fraction underwent reduction and alkylation [30,31]. Dithiothreitol was added to 10 mM and incubated at 37 °C for 4 h, followed by iodoacetamide at 50 mM for 40 min in the dark. After desalting with self-packed columns, samples were vacuum-dried and reconstituted for peptide identification by Biotech Pack Scientific Co., Ltd. (Beijing, China). Liquid chromatography (LC) was performed using a Ultimate 3000 system (Thermo Fisher Scientific, Waltham, MA, USA) with an Acclaim PepMap RPLC C18 trap column (300 μm × 5 mm, 5 μm, 100 Å) and analytical column (75 μm × 150 mm, 3 μm, 100 Å). The mobile phase included Solvent A (0.1% formic acid, 2% acetonitrile) and Solvent B (0.1% formic acid, 80% acetonitrile), at a flow rate of 300 nL/min. The 78 min gradient elution was as follows: 6% B at 0 min, ramped to 9% (0–8 min), 14% (8–24 min), 30% (24–60 min), 40% (60–75 min), and 95% (75–78 min). Mass spectrometry (MS) was conducted using a Orbitrap Elite™ Hybrid Ion Trap-Orbitrap Mass Spectrometer (Thermo Fisher Scientific, USA) with an electrospray ionization (ESI) source in full MS and MS/MS modes. Instrument parameters are listed in Table 1.

### 2.5. Absolute Quantification of Active Peptides

Absolute quantification of active peptides was performed using parallel reaction monitoring (PRM). Sample pretreatment involved preliminary peptide quantification using the Pierce Quantitative Colorimetric Peptide Assay, followed by the addition of 50 mM NH_4_HCO_3_ buffer to a final volume of 100 μL. Dithiothreitol was then added to a final concentration of 10 mM, and the mixture was incubated in a 37 °C water bath for 3 h for reduction. Subsequently, iodoacetamide was added to a final concentration of 50 mM and incubated in the dark for 40 min for alkylation. Treated peptides (15 μg) were desalted using custom-packed columns, vacuum-dried at 45 °C, and redissolved. Heavy isotope-labeled standard peptides corresponding to the target sequences were synthesized, labeled at the leucine (L) residue. All PRM sample preparation and mass spectrometric analysis were performed by Biotech Pack Scientific Co., Ltd. (Beijing, China).

PRM analysis was carried out under the same chromatographic gradient and conditions as previously described, using a 150 μm × 150 mm column with 1.9 μm particles. Mass spectrometry was performed in a positive ion mode with a collision voltage of 27 V. The precursor ion information is provided in Table 2. Samples were dissolved in 0.1% formic acid and 2% acetonitrile, vortexed, and centrifuged at 13,200 rpm for 10 min, and the supernatant was injected into the LC-MS/MS system as described in Section 2.4. A calibration curve was generated using synthetic heavy isotope-labeled peptides (10–5000 fmol). Each standard was mixed 1:1 with peptide digest, diluted to the same volume, and analyzed. The calibration curve was constructed by plotting peptide concentration (x-axis) against peak area (y-axis).

Data were analyzed using Skyline software (version 23.1.0.380; MacCoss Lab, University of Washington, Seattle, WA, USA) with a cut-off of 0.99. The five most intense fragment ions per peptide were manually integrated, and the top three used for quantification: if all three had relative standard deviations (RSDs) < 20%, their average was used; if only two met the threshold, their average was taken; if none met the threshold, the most intense single fragment ion was used for quantification.

### 2.6. Peptide Activity Verification and Dose-Dependent Analysis of Synthetic Peptides

Candidate peptide sequences identified by LC-MS/MS were synthesized using GenScript Biotech (Nanjing, China). Peptide solubility was tested in various solvents to determine optimal conditions for functional assays. Initial screening was performed in 30 mL of inorganic salt medium with 200 mg/L L-tryptophan. *A. pascens* ZZ21 was inoculated at 1% (*v*/*v*), and peptides were tested at 1 ppm and 0.1 ppm. The peptides were dissolved according to their solubility profiles, with corresponding solvent controls: CK-A (1 ppm blank) and CK-B (0.1 ppm blank). Cultures were incubated at 30 °C, 250 rpm for 48 h, after which IAA levels in the supernatants were measured.

Based on preliminary results, peptides with potential bioactivity were selected for further validation and dose-dependent assays. The experimental setup remained the same but included a wider concentration range. Each treatment had five biological replicates. IAA was quantified after 48 h to evaluate dose-dependent effects and consistency in growth promotion.

### 2.7. Statistical Analysis

All data were analyzed using SPSS 22.0 (IBM, Armonk, NY, USA), and graphs were created with Origin 8.5 (OriginLab, Northampton, MA, USA). Data are expressed as mean ± standard error (SE). Data distributions and homogeneity of variances were checked using the Shapiro–Wilk test and Levene’s test, respectively. Significant differences (*p* < 0.05) were determined by Duncan’s multiple-range test, Dunnett’s test, or an independent-sample *t*-test, as specified in the figure legends. In the figures, statistical significance is indicated by different letters (a, b) or asterisks (*).

## 3. Results

### 3.1. Peptides in C. elegans Exudates Enhance IAA Production by A. pascens ZZ21

**Figure 1:**
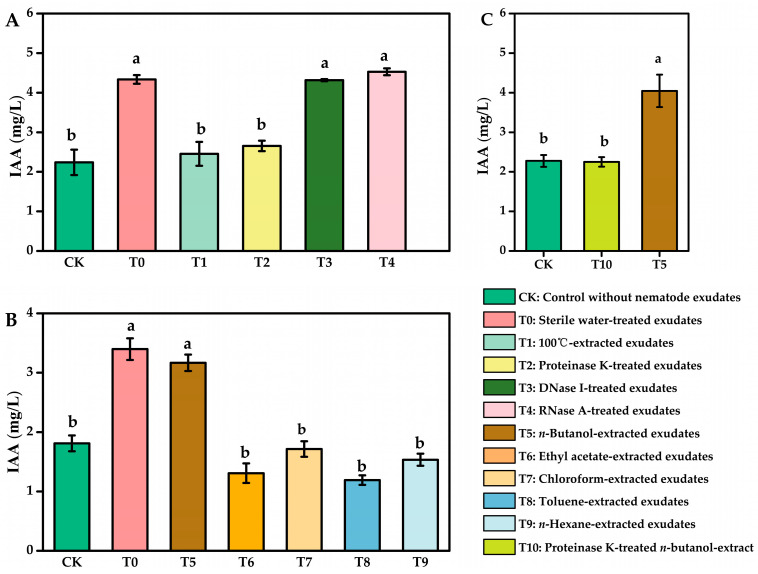
Characterization of active components in *C. elegans* exudates that promote IAA production by *A. pascens* ZZ21: (**A**) effects of enzymatic and heat treatments on IAA-promoting activity; (**B**) IAA-inducing activity of exudate extracts obtained using different organic solvents; (**C**) effect of protease K treatment on the n-butanol extract bioactivity. Data are expressed as mean ± SE. Values with different letters (a, b) are significantly different as determined by Duncan’s multiple-range test at *p* < 0.05.

Based on previous findings [25], *C. elegans* N2 exudates significantly enhanced IAA production by the plant growth-promoting bacterium *A. pascens* ZZ21. This study further investigated the properties and stability of the active components in *C. elegans* exudates using a series of treatments. Heat treatment (100 °C, 10 min) significantly reduced the exudates’ stimulatory effect on IAA production, lowering IAA levels to near those of the control (CK) (Figure 1A), indicating heat sensitivity of the active compound. Similarly, proteinase K treatment significantly diminished activity, reducing IAA production enhancement from 93.65% to 18.59% (Figure 1A), suggesting the active component is likely a protein or peptide susceptible to proteolysis. In contrast, DNase I and RNase A treatments had no effect (Figure 1A), ruling out nucleic acids as essential for stimulation. To assess the physicochemical properties of the active compounds, nematode exudates were extracted with five organic solvents of varying polarity—n-butanol, ethyl acetate, chloroform, toluene, and n-hexane. These solvents were selected based on their dielectric constants, which reflect solvent polarity, decreasing in the following order: n-butanol > ethyl acetate > chloroform > toluene > n-hexane. Among them, only the n-butanol extract retained stimulatory activity comparable to the untreated control (T0), while the other extracts showed no effect (Figure 1B). Since n-butanol precipitates large proteins, the active substance is unlikely to be a high-molecular-weight protein but rather a small, polar, hydrophilic peptide, as it was not extractable by lipophilic or hydrophobic solvents. Further treatment of the n-butanol extract with proteinase K abolished its activity (Figure 1C), with IAA production dropping to levels indistinguishable from the blank control. These results suggest the active component in *C. elegans* exudates is most likely a highly polar peptide.

### 3.2. Identification of Bioactive Peptides in C. elegans Exudates

To identify the active compounds, the n-butanol extract was analyzed via liquid chromatography–tandem mass spectrometry (LC-MS/MS), yielding 30 reliable peptide sequences via manual interpretation (Table 3; MS/MS data in Figures S2–S31). Specifically, de novo sequencing was performed on high-resolution MS/MS data, followed by manual inspection of selected spectra based on ion series continuity, fragment ion accuracy, and peptide abundance to confirm sequence reliability. This analysis was conducted by Biotech Pack Scientific Co., Ltd. (Beijing, China). The identified peptides had molecular weights of 700–1400 Da, comprising 7–14 amino acids, and were primarily linear with minimal modifications. All 30 candidate peptides were synthesized (Nanjing GenScript Biotech Co., Ltd., Nanjing, China), and their solubility was assessed (Table S1). Notably, 18 peptides (P1–P11, P15–P17, P19, P21, P23, and P26) were water-soluble, while 12 exhibited solubility < 0.1 mg/mL in water. Among these, P30 was soluble (≤5 mg/mL) in PBS, and the remaining 11 required DMSO for dissolution.

### 3.3. Activity Validation of Synthetic Peptides

**Figure 2:**
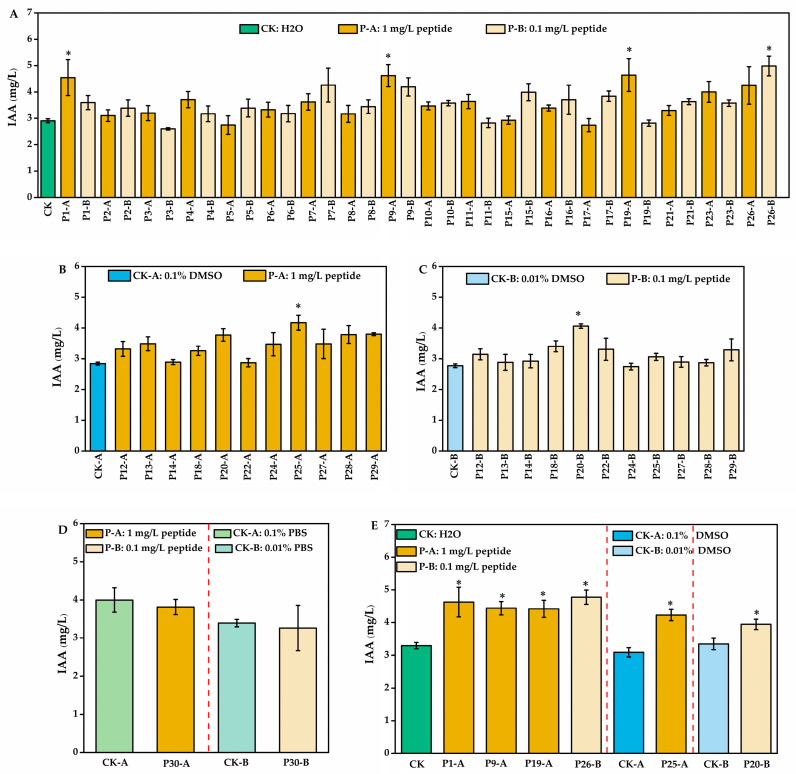
Activity validation of synthetic peptides promoting IAA production in *A. pascens* ZZ21: (**A**) effects of 18 water-soluble peptides on IAA production; (**B**,**C**) effects of 11 DMSO-soluble peptides on IAA production at (**B**) 1 mg/L and (**C**) 0.1 mg/L; (**D**) effect of PBS-soluble peptide on IAA production; (**E**) effects of six selected peptides in replicated validation assays. In panels (**D**,**E**), dashed lines are used to visually separate results from distinct experiments. Data are expressed as mean ± SE. Asterisks indicate significant differences at *p* < 0.05, as determined by Dunnett’s test or independent-sample *t*-test.

**Figure 3:**
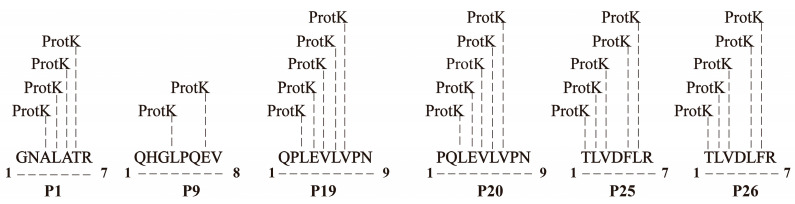
Predicted proteinase K cleavage sites in the six bioactive peptides (P1, P9, P19, P20, P25, and P26), based on PeptideCutter analysis (ExPASy). Vertical dashed lines labeled “ProtK” indicate predicted cleavage positions by proteinase K, occurring to the right side (C-terminal side) of the marked amino acids. Horizontal dashed lines represent the full-length linear peptide sequences, and the numbers at both ends indicate the position of the first and last amino acid residues.

Based on solubility differences, the 30 candidate peptides were grouped and screened for their ability to promote IAA production in *A. pascens* ZZ21 (Figure 2). Among the water-soluble peptides, P1, P9, and P19 increased IAA synthesis by 56.96%, 58.89%, and 59.57%, respectively, at 1 mg/L, while P26 enhanced production by 71.26% at 0.1 mg/L (Figure 2A). Among the 11 DMSO-soluble peptides, P20 and P25 significantly increased IAA levels by 46.35% and 46.68% at 0.1 mg/L and 1 mg/L, respectively (Figure 2B,C). In contrast, P30 (PBS-soluble) showed no notable bioactivity (Figure 2D). Six peptides (P1, P9, P19, P20, P25, and P26) demonstrating promising effects in initial screening were selected for repeated validation. All six peptides consistently promoted IAA synthesis to varying degrees, with high reproducibility (Figure 2E). Further analysis of proteinase K recognition sites revealed that each peptide contained potential cleavage sites (Figure 3), explaining their loss of bioactivity after proteinase K treatment and confirming their role as the primary bioactive components.

### 3.4. Dose-Dependent Effects of Active Peptides on IAA Production

**Figure 4:**
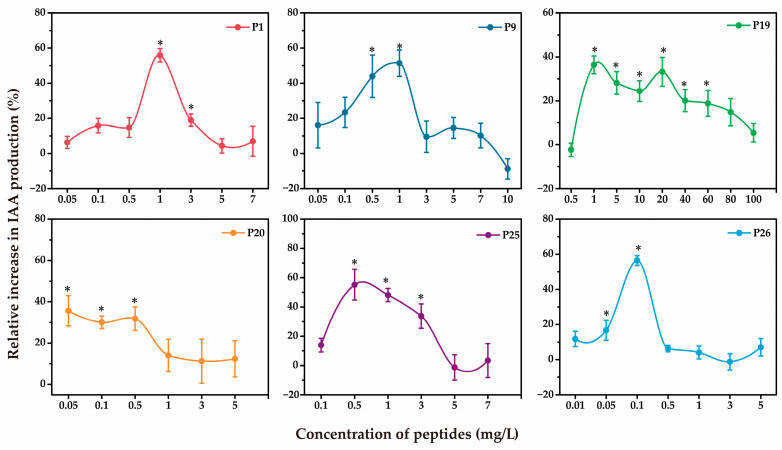
Dose-dependent curves of six peptides showing their effects on IAA production by *A. pascens* ZZ21. Data are expressed as mean ± SE. Asterisks indicate significant differences at *p* < 0.05, as determined by Dunnett’s test.

To evaluate dose-dependent effects, six bioactive short peptides (P1, P9, P19, P20, P25, and P26) were tested across concentration gradients (Figure 4). P1, P9, P25, and P26 exhibited bell-shaped response curves, showing the highest bioactivity at 1 mg/L, 1 mg/L, 0.5 mg/L, and 0.1 mg/L, with corresponding IAA production increases of 55.95%, 51.4%, 55.16%, and 56.44%, respectively. However, their stimulatory effects declined at higher concentrations. P19 showed a broad effective range (1–60 mg/L), sustaining IAA stimulation across all concentrations. In contrast, P20 lacked a clear dose-dependent trend, suggesting it may regulate IAA synthesis through a distinct regulatory mechanism compared to the other peptides.

### 3.5. Quantitative Analysis of Candidate Bioactive Peptides

To quantify nematode-secreted peptides in the experimental system, we used PRM to measure six candidate short peptides (P1, P9, P19, P20, P25, and P26). These exudates were extracted with n-butanol, dried under nitrogen, and reconstituted in sterile water. A linear standard curve was generated using heavy isotope-labeled peptide standards, with concentration (x-axis) plotted against peak area (y-axis) (Table S2). Peptide concentrations were calculated based on molecular weights. During standard curve construction, the heavy-labeled P20 peptide’s peak area lacked a linear relationship with concentration, so P20 was excluded from quantification. The remaining five peptides were detected in exudate samples at concentrations of 9.94 × 10^−2^ to 3.92 × 10^−1^ ng/μL (Table 4). Normalized to nematode abundance, secretion rates per *C. elegans* individual ranged from 6.63 × 10^−3^ to 2.61 × 10^−2^ ng, with P9 being the most abundant and P26 the least. By scaling the sample volume to the total bacterial culture volume, we estimated the theoretical final peptide concentrations of these five peptides in the bacterial culture system to be 1.33 × 10^−2^ to 5.22 × 10^−2^ μg/mL.

## 4. Discussion

### 4.1. Nematode Peptides as Potent Promoters of Bacterial IAA Synthesis

As early as the 1980s and 1990s, numerous studies reported the presence of neuropeptides in free-living nematodes. Among them, the Phe-Met-Arg-Phe-NH2 type neuropeptide was found to be widespread across various nematodes and to play a key regulatory role in their physiology and biochemistry [32,33,34,35]. In recent years, research has shifted toward peptides secreted by parasitic nematodes [12,36,37]. Homogenates or exudates from plant–parasitic nematodes contain small peptides (molecular weight < 3 kDa) that resemble endogenous plant peptides in function and are sensitive to heat and proteases. These peptides can induce plant cell mitosis, thereby promoting growth and development. Subsequent studies confirmed their role as plant cytokinins [38,39]. Plant–parasitic nematodes secrete hormone-like molecules such as cytokinins to manipulate host cell division and differentiation, enabling long-term parasitism and complex host–parasite interactions [40,41]. In contrast, systematic research and mechanistic insights into how exudates from free-living soil nematodes influence microbial activity and plant growth remain lacking.

Previous research showed that *C. elegans* exudates significantly enhanced IAA production by the plant growth-promoting bacterium *A. pascens* ZZ21 [25]. Building on this, the present study further characterized the physicochemical and functional properties of the active components. Heat and enzymatic treatments indicated that the activity was highly sensitive to high temperature and proteinase K but unaffected by DNase or RNase, suggesting the active factors are likely small peptides or proteins, not nucleic acids. Based on the principle of “like dissolves like,” five organic solvents of varying polarity were tested. Among them, n-butanol—a medium-polarity solvent combining properties of long- and short-chain alcohols—proved most effective. While insoluble in water, it can dissolve polar compounds soluble in short-chain alcohols and also precipitate large proteins. The n-butanol extract retained IAA-promoting activity, indicating that the active component is likely a small, proteinase K-sensitive peptide rather than a larger protein. LC-MS/MS analysis of the extract identified 30 linear short peptides (700–1400 Da, 7–14 amino acids). Notably, further analysis revealed that these peptides consistently displayed a distinct profile: they were short and predominantly terminated in a basic lysine (K) or arginine (R) residue. This profile is precisely the characteristic signature of mature, bioactive neuropeptides generated through endogenous biological processing. This established biosynthetic pathway involves the cleavage of larger, inactive precursor proteins by endogenous proprotein convertases at specific recognition sites, which typically consist of single or dibasic motifs of basic amino acids like lysine and arginine [42,43]. Among them, six synthetic peptides (P1, P9, P19, P20, P25, and P26) showed significant IAA-enhancing activity in initial screening, suggesting they are key active components. All six had predicted proteinase K cleavage sites, consistent with loss of activity upon protease treatment. Further dose–response experiments revealed varied activity patterns: P1, P9, P25, and P26 showed bell-shaped response curves, with peak activity at specific concentrations and reduced effects at higher doses. Biologically, this pattern is often attributed to factors such as receptor saturation, agonist-induced receptor desensitization, or the activation of negative feedback loops at high ligand concentrations [44,45,46,47]. P19 maintained consistent activity over a wide range, indicating a more robust regulatory mechanism, while P20 showed no clear dose dependence, implying a distinct mode of action. PRM quantified these peptides at low concentrations in nematode exudates (9.94 × 10^−2^–3.92 × 10^−1^ ng/μL), yet they exhibited strong bioactivity in promoting IAA synthesis by *A. pascens* ZZ21. This suggests they may act synergistically in natural environments when co-existing. The presence of multiple effective concentration ranges further indicates that a single peptide can remain bioactive across variable levels, supporting stable bacterial modulation under fluctuating environmental conditions.

While the mechanism remains to be clarified, these peptides may exert their effects via the IPyA pathway, as observed for the crude extract in previous work. However, it is unclear whether they act exclusively through this route. Given that bacterial IAA biosynthesis is finely regulated by diverse external cues [48,49,50], these peptides may be sensed by membrane-bound receptors and initiate intracellular signaling that promotes auxin production.

### 4.2. Ecological Significance and Future Perspectives of Nematode Peptides

Although nematodes are known to secrete a variety of signaling small molecules, current research has primarily focused on ascarosides. These fatty acid-derived compounds not only regulate nematode behavior and development but are also recognized by plants, fungi, bacteria, and even mammals, mediating complex cross-kingdom communication networks [51,52,53,54]. In addition, nematode exudates can attract or repel specific bacterial taxa, thereby influencing microbial community dynamics and facilitating nematode adaptation to variable environmental conditions [55,56].

Building on this foundation, the present study reveals that bacterivorous nematodes can promote IAA production in rhizosphere plant growth-promoting bacteria by secreting specific small peptides, suggesting a previously unrecognized ecological regulatory mechanism. We propose that the function of these peptide-based exudates likely extends beyond modulating specific microbial “prey.” Similar to how plant root exudates can systematically reshape the rhizosphere microbiome [57,58,59,60], nematode-derived peptides may also act as infochemicals, influencing a broad spectrum of rhizosphere microorganisms, including non-target bacteria, fungi, and microalgae. Moreover, these signals may participate in regulating key microbial processes such as nitrogen fixation, phosphate solubilization, and mycorrhizal symbiosis [61,62]. By shaping multi-kingdom microbial consortia composed of bacteria, fungi, and microalgae, nematodes may create a stable, mutualistic, and ecologically resilient microenvironment, thereby enhancing their survival and adaptability in complex soil ecosystems.

These findings suggest that nematode-derived peptides may serve as in situ biostimulants by modulating beneficial microbial functions. Unlike conventional methods that introduce exogenous microbes, this approach leverages native communities, aligning with sustainable agriculture goals. Nevertheless, issues such as peptide stability in soil and large-scale production remain unresolved. Further research should address formulation, mode of action, and field efficacy to support their practical application.

## 5. Conclusions

This study identified six small peptides secreted by the free-living nematode *C. elegans*, which significantly promoted IAA synthesis in *A. pascens* ZZ21. These peptides showed diverse dose–response patterns. Despite their low concentrations in the nematode exudates, they exhibited strong bioactivity, suggesting localized accumulation or synergistic effects. This finding deepens our understanding of the chemical ecology of free-living nematodes and provides new insights into their potential roles in regulating rhizosphere microbial activity. We propose that nematode-derived peptides function as key signaling molecules within the plant–microbe–nematode interaction network by modulating the metabolism of plant growth-promoting bacteria, thus advancing our knowledge of belowground interkingdom communication.

## Figures and Tables

**Table 1:** Parameters for the mass spectrometry analysis of peptides.

Parameter Category	Parameter	Setting/Value
Full MS Scan (MS1)	Mass Resolution	70,000
	Automatic Gain Control (AGC) Target	3 × 10^6^
	Maximum Injection Time (IT)	40 ms
	Scan Range	350 to 1800 *m*/*z*
MS/MS Scan (dd-MS2)	Mass Resolution	75,000
	Automatic Gain Control (AGC) Target	1 × 10^5^
	Maximum Injection Time (IT)	60 ms
	TopN (Number of Precursors for Fragmentation)	60
	Normalized Collision Energy (NCE)	27

**Table 2:** Target peptides and their parameters for PRM quantification.

Peptides	*m*/*z*	Start Time (min)	End Time (min)	Charge
GNAL*ATR	355.21	5	20	2
QHGL*PQEV	457.74	10	35	2
QPL*EVL*VPN	511.81	40	70	2
TL*VDFL*R	439.27	50	75	2
TL*VDL*FR	439.27	55	70	2
GNALATR	351.70	5	20	2
QHGLPQEV	454.24	10	35	2
QPLEVLVPN	504.79	40	70	2
TLVDFLR	432.25	50	75	2
TLVDLFR	432.25	55	70	2

Note: L* represents leucine labeled with stable heavy isotopes.

**Table 3:** Mass spectrometry identification of 30 candidate peptides.

No.	Peptides	Charge	*m*/*z*	Peptide Mass
P1	GNALATR	2	351.70	701.38
P2	GPGPVADYDPGLAR	3	462.23	1383.68
P3	SSYQYKDPGLAR	3	462.23	1383.68
P4	SSQYYKDPGLAR	3	462.23	1383.68
P5	GSAVAVGR	3	358.71	715.40
P6	AEFAEVSK	2	440.72	879.43
P7	VVTDSFR	2	412.22	822.42
P8	LVSELTK	2	395.24	788.46
P9	QHGLPQEV	2	454.24	906.46
P10	ALADDFR	2	404.20	806.39
P11	DSGLVLK	2	366.22	730.42
P12	TLALAFGLTA	2	489.29	976.56
P13	LVLQLDNAK	2	507.30	1012.59
P14	LYYELAR	2	464.25	926.49
P15	FASFLDK	2	414.22	826.42
P16	NLTLSNPSDGLTSTTPNPK	2	979.00	1955.98
P17	TTPHNLDVLE	2	569.79	1137.57
P18	FLDLSLK	2	418.25	834.49
P19	QPLEVLVPN	2	504.79	1007.57
P20	PQLEVLVPN	2	504.79	1007.57
P21	QPLVELVPN	2	504.79	1007.57
P22	PQLELVVPN	2	504.79	1007.57
P23	LVVNGSAALGL	2	507.30	1012.59
P24	VLQAASAALGL	2	507.30	1012.59
P25	TLVDFLR	2	432.25	862.49
P26	TLVDLFR	2	432.25	862.49
P27	LTVDLFR	2	432.25	862.49
P28	VTDVSEVFFK	2	585.81	1169.60
P29	EPTSVNLLAE	2	536.78	1071.54
P30	EPTSVGVALAE	2	536.78	1071.54

**Table 4:** Quantified concentrations of candidate peptides and their estimated levels in culture.

No.	Peptides	Concentration of Peptides (ng/μL)	Peptide Content per Nematode (ng/worm)	Concentration of Peptides in Medium (μg/mL)
P1	GNALATR	(3.50 ± 0.07) × 10^−1^	(2.33 ± 0.04) × 10^−2^	(4.66 ± 0.09) × 10^−2^
P9	QHGLPQEV	(3.92 ± 0.06) × 10^−1^	(2.61 ± 0.04) × 10^−2^	(5.22 ± 0.07) × 10^−2^
P19	QPLEVLVPN	(2.49 ± 0.34) × 10^−1^	(1.66 ± 0.23) × 10^−2^	(3.32 ± 0.46) × 10^−2^
P25	TLVDFLR	(1.80 ± 0.06) × 10^−1^	(1.20 ± 0.04) × 10^−2^	(2.40 ± 0.08) × 10^−2^
P26	TLVDLFR	(9.94 ± 1.68) × 10^−2^	(6.63 ± 1.10) × 10^−3^	(1.33 ± 0.22) × 10^−2^

Note: Data are presented as mean ± SE from three independent biological replicates (*n* = 3).

## Data Availability

The original contributions presented in this study are included in the article/Supplementary Material. Further inquiries can be directed to the corresponding authors.

## Supplementary Materials

The following supporting information can be downloaded at: https://www.mdpi.com/article/10.3390/microorganisms13081951/s1, Figure S1: Anatomy of *Caenorhabditis elegans*; Figures S2–S31: Mass spectra of the 30 peptide sequences; Table S1: Solubility assessment of 30 candidate peptides; Table S2: The standard curves of P1, P9, P19, P25, and P26.
