# Supplementary material for: Identification of Bioactive Peptides from Caenorhabditis elegans Secretions That Promote Indole-3-Acetic Acid Production in Arthrobacter pascens ZZ21
Source: Microorganisms. 2025 Aug 21;13(8):1951. doi: 10.3390/microorganisms13081951 (PMC12388801; doi:10.3390/microorganisms13081951)
Supplement: Supplementary file 1 [file microorganisms-13-01951-s001.zip › Supplementary Figures.pdf]

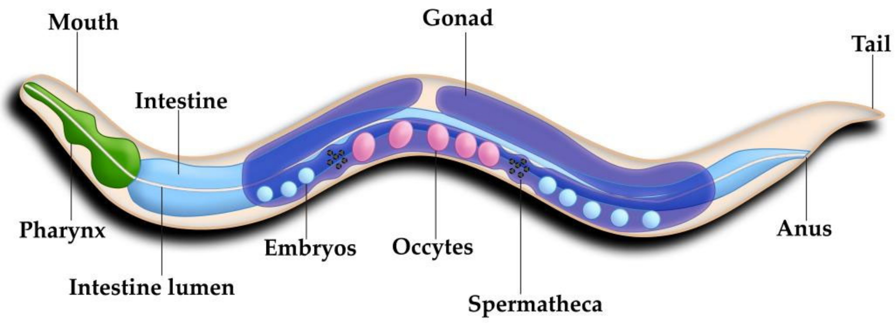

Figure S1: Anatomy of *Caenorhabditis elegans*

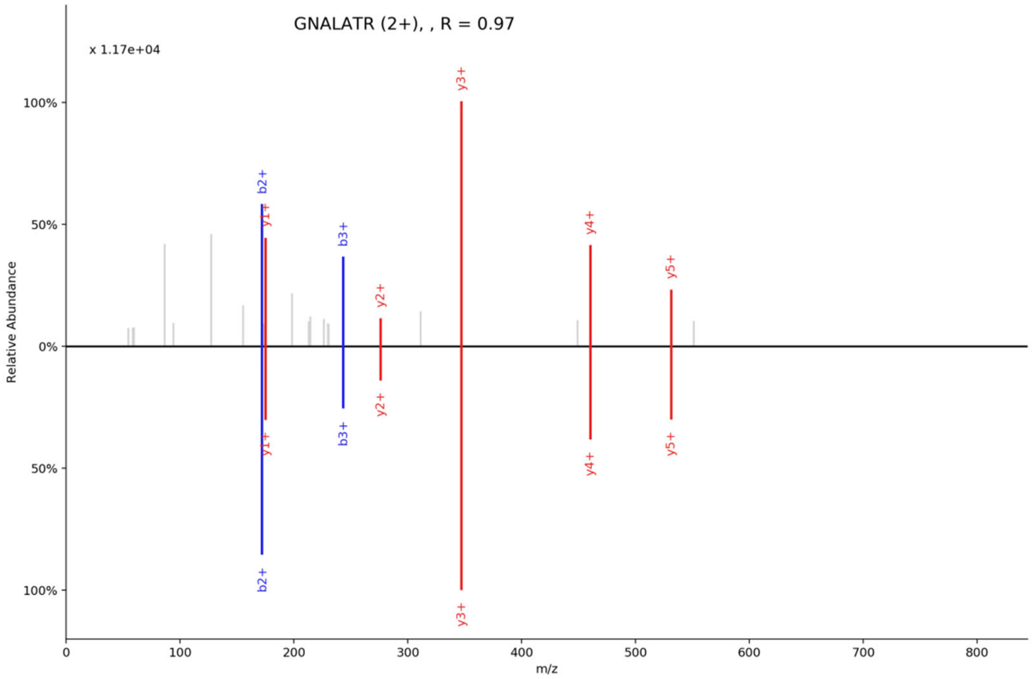

Figure S2. Mass spectrum of peptide P1

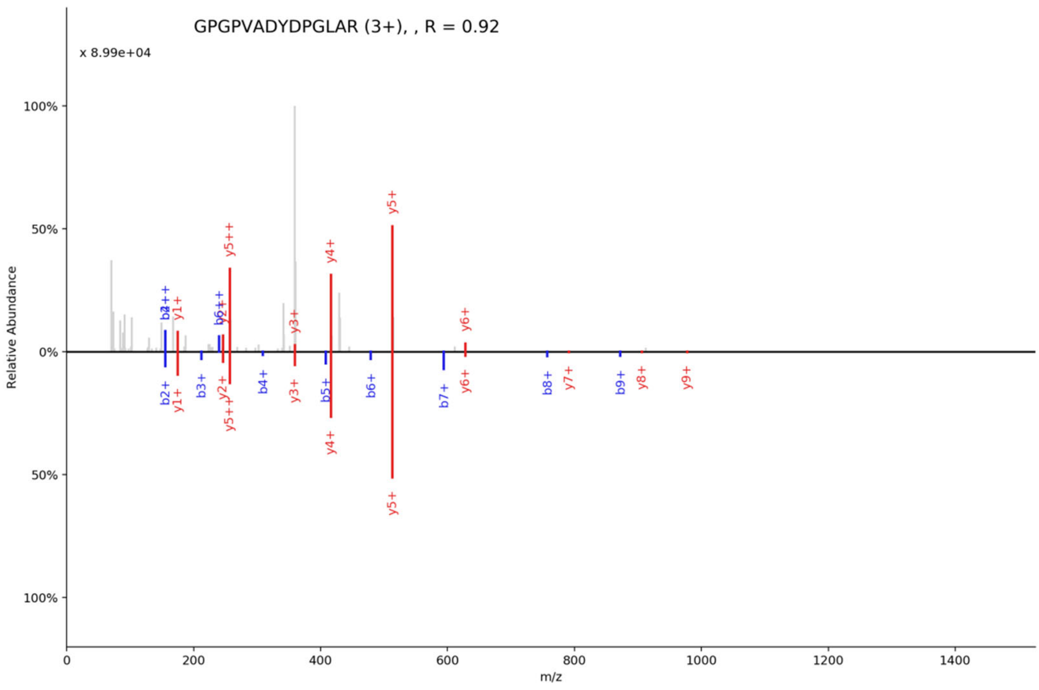

Figure S3. Mass spectrum of peptide P2

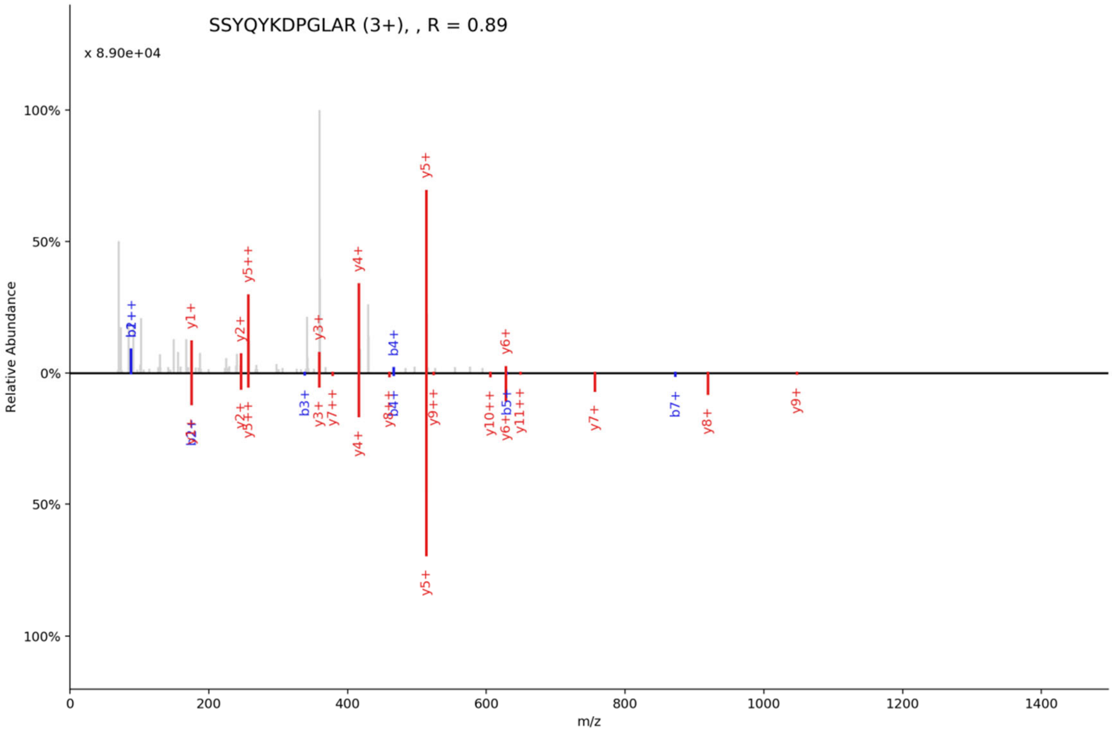

Figure S4. Mass spectrum of peptide P3

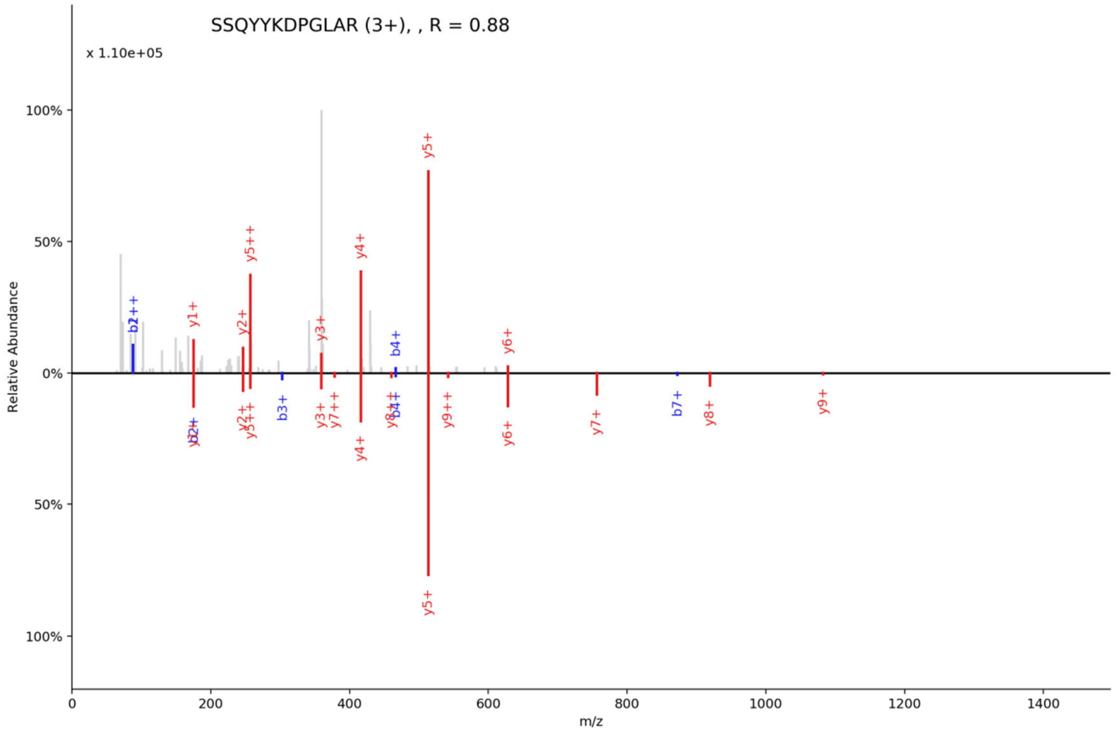

Figure S5. Mass spectrum of peptide P4

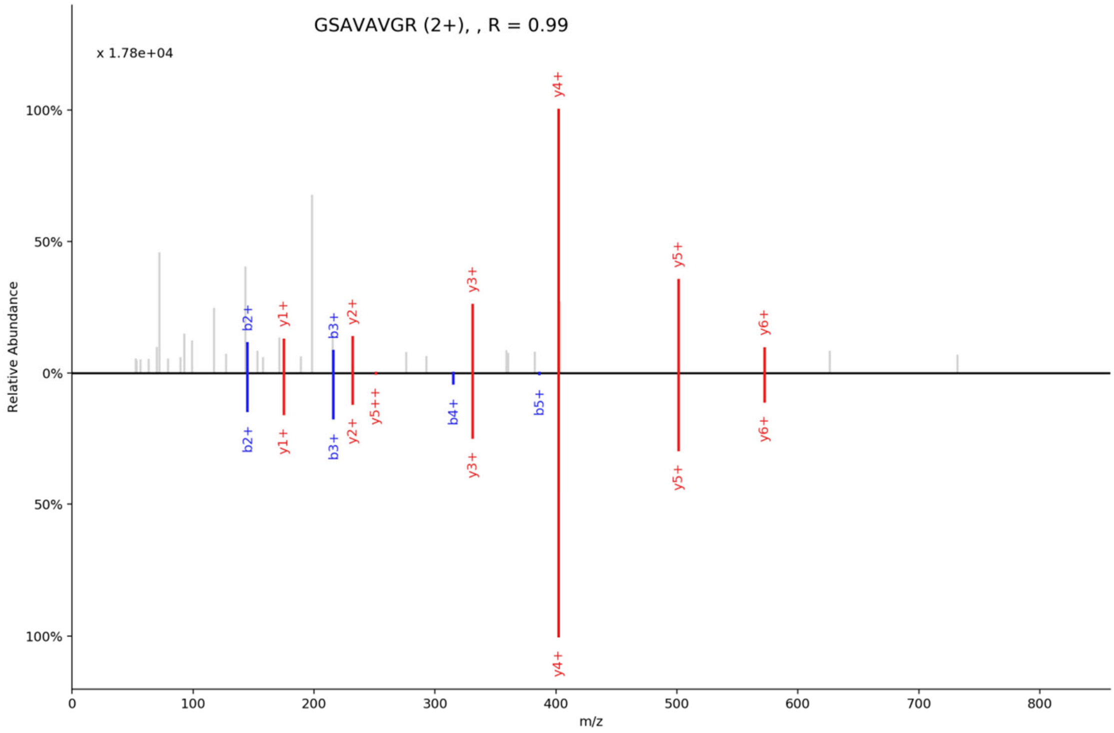

Figure S6. Mass spectrum of peptide P5

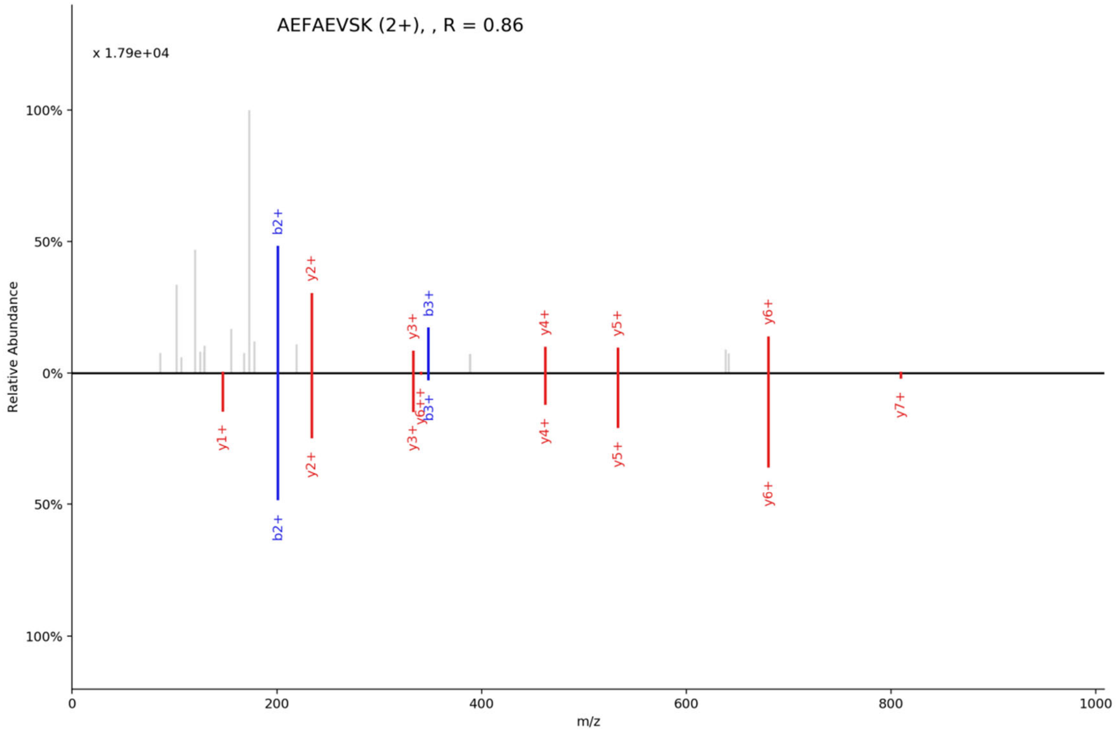

Figure S7. Mass spectrum of peptide P6

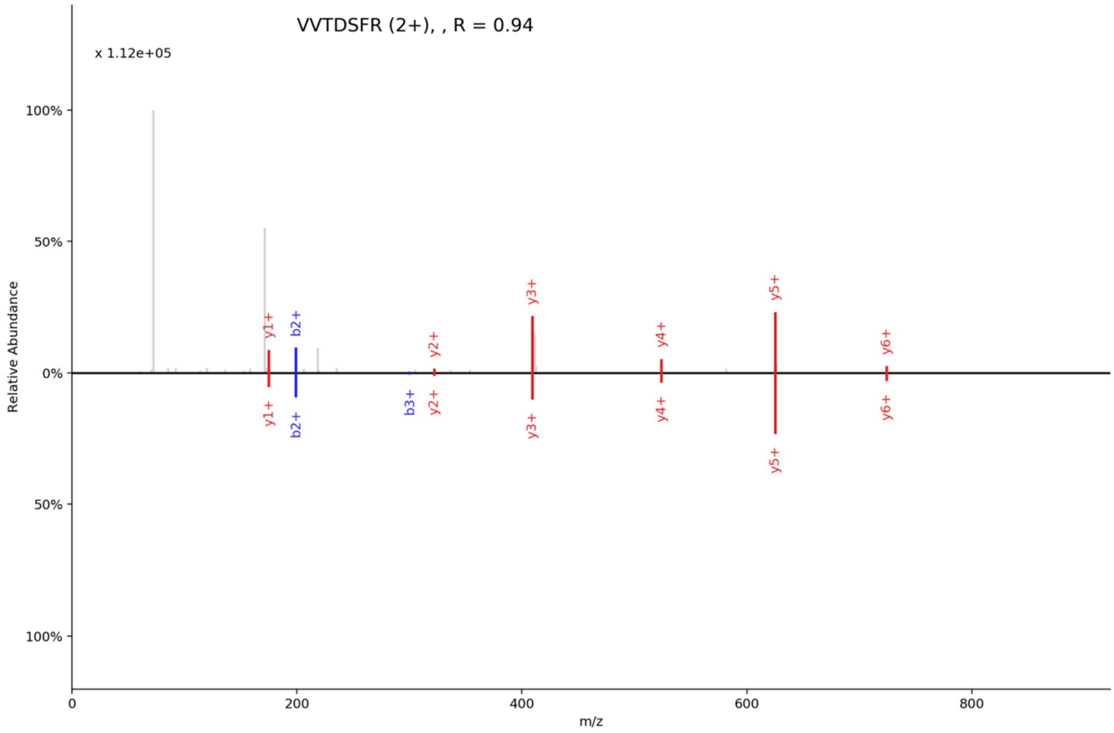

Figure S8. Mass spectrum of peptide P7

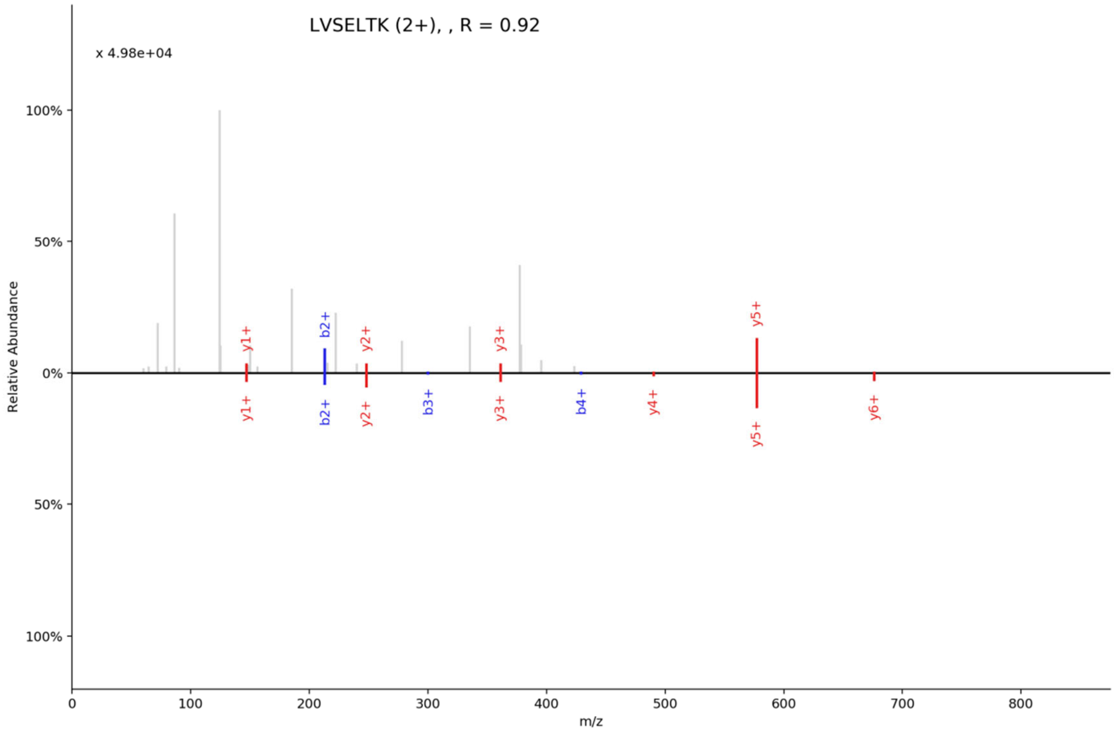

Figure S9. Mass spectrum of peptide P8

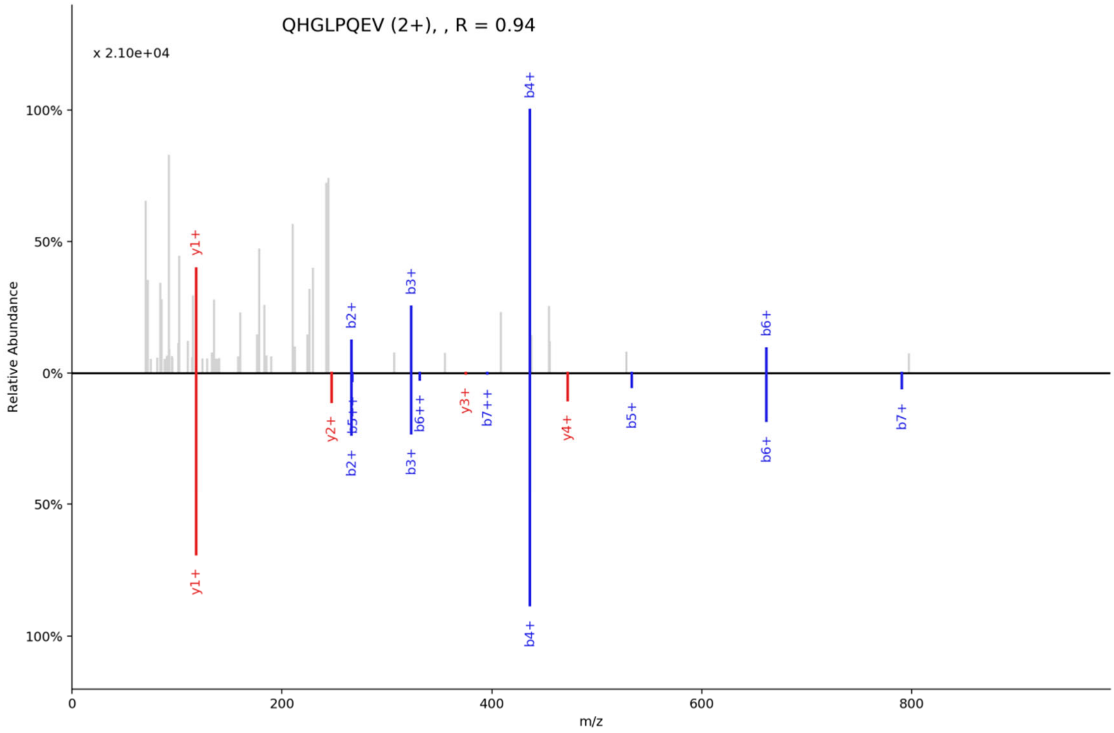

Figure S10. Mass spectrum of peptide P9

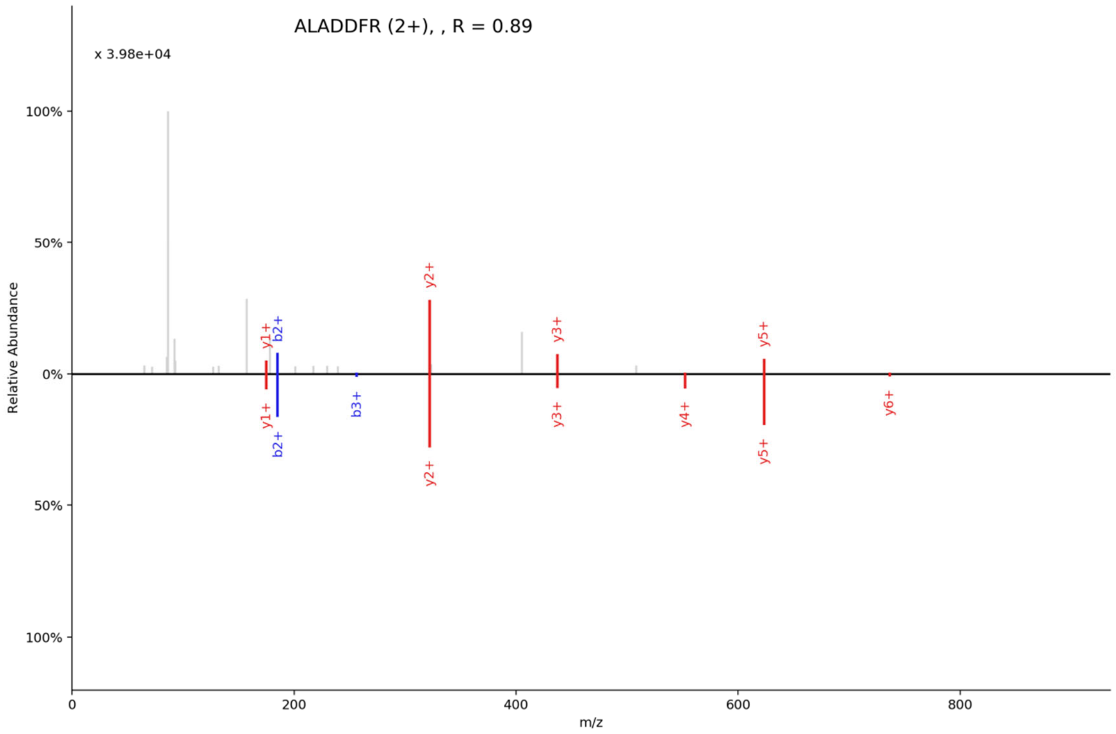

Figure S11. Mass spectrum of peptide P10

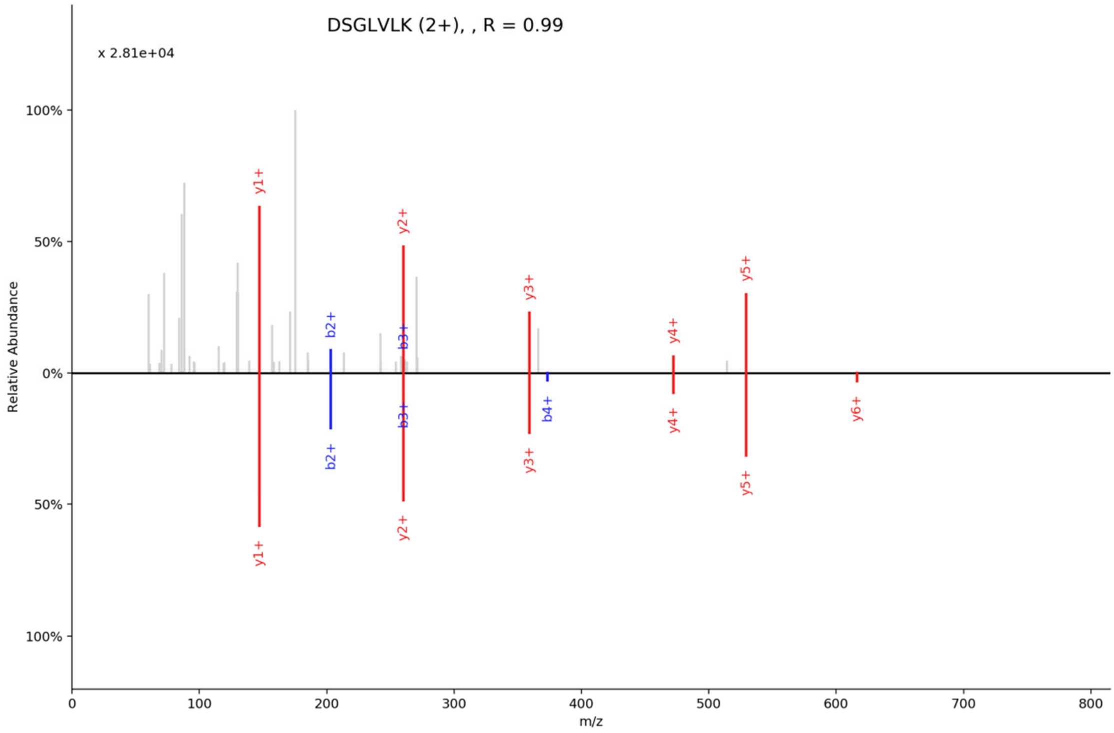

Figure S12. Mass spectrum of peptide P11

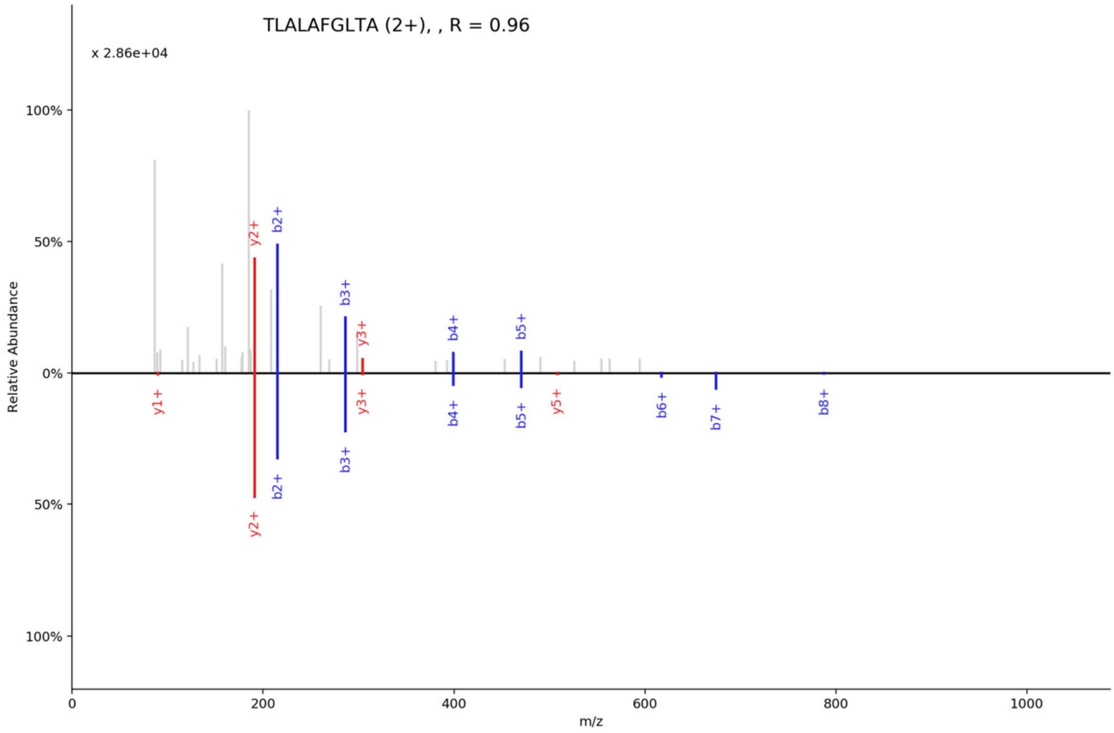

Figure S13. Mass spectrum of peptide P12

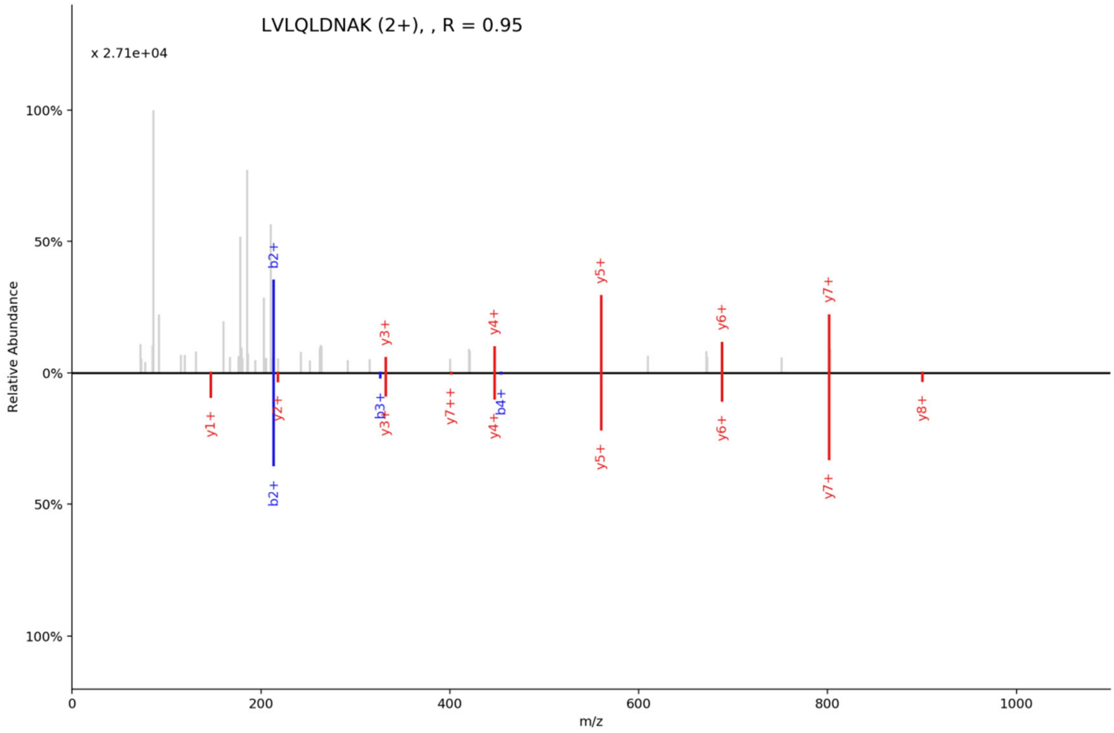

Figure S14. Mass spectrum of peptide P13

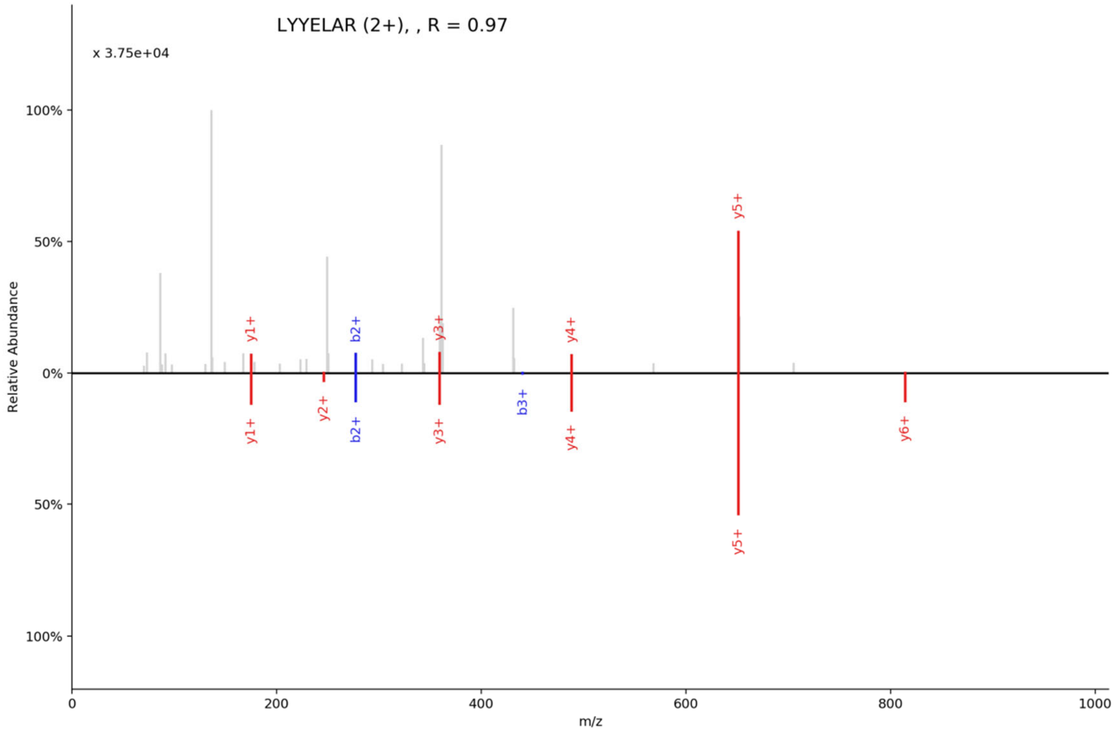

Figure S15. Mass spectrum of peptide P14

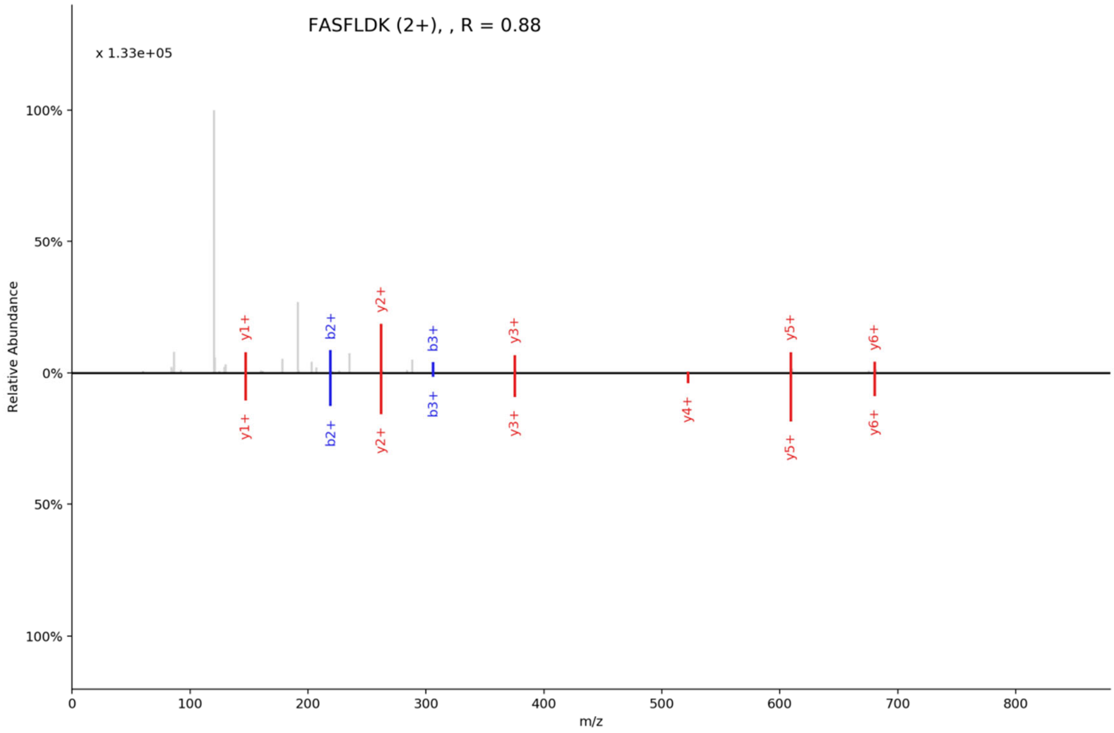

Figure S16. Mass spectrum of peptide P15

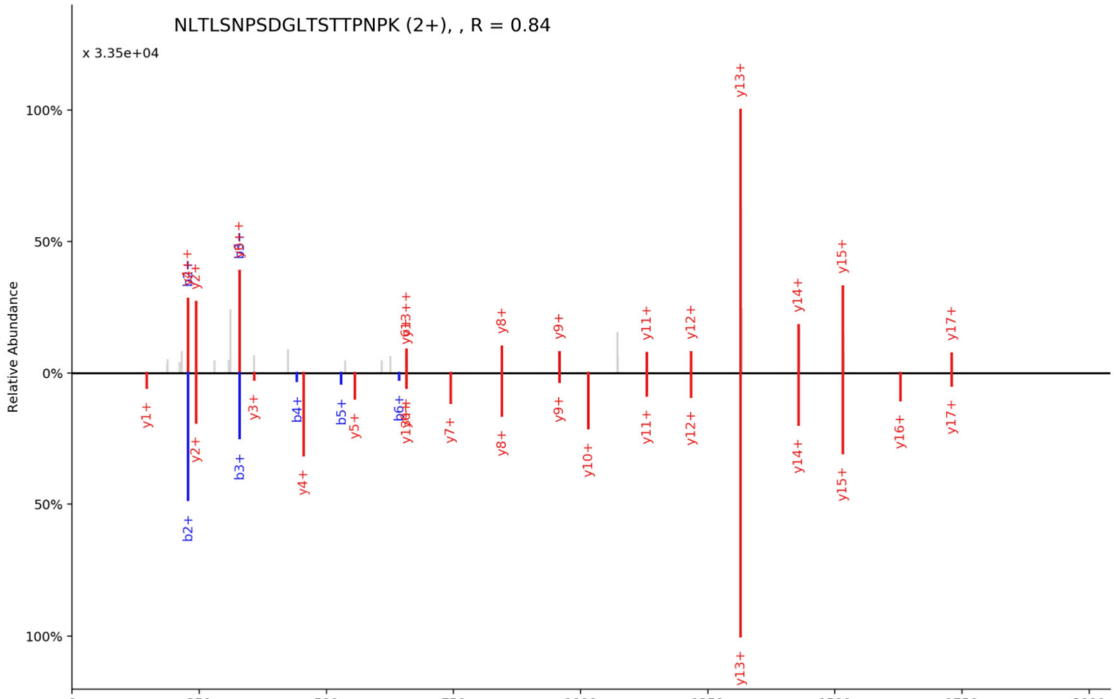

Figure S17. Mass spectrum of peptide P16

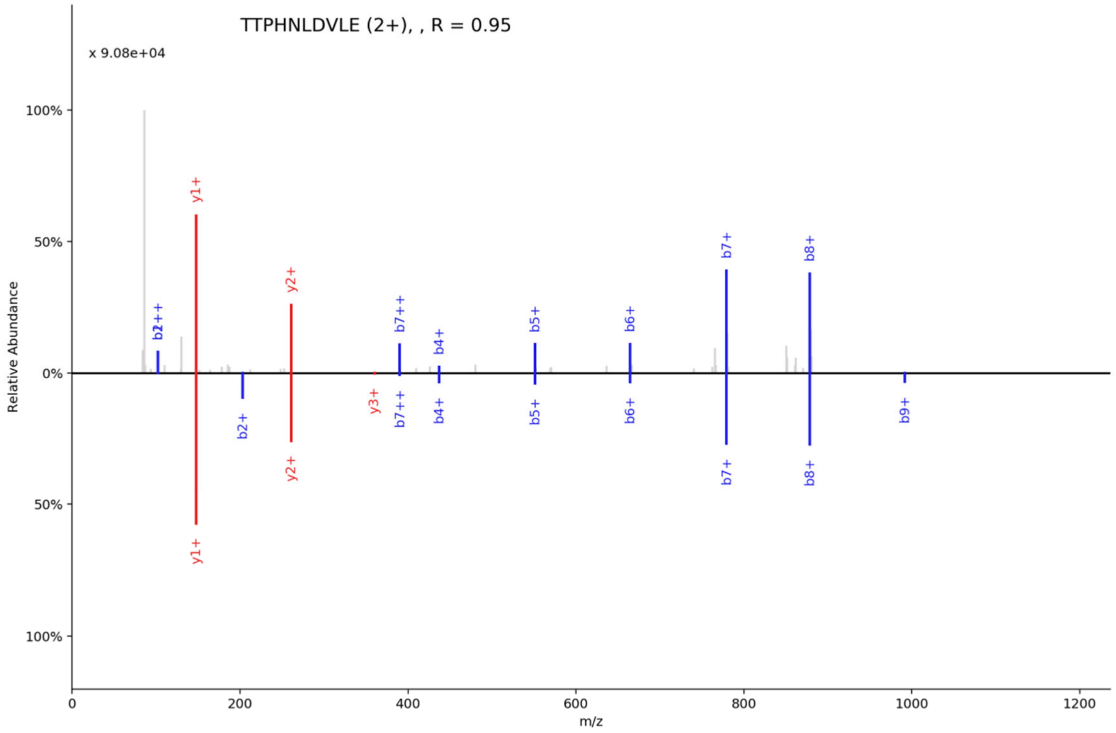

Figure S18. Mass spectrum of peptide P17

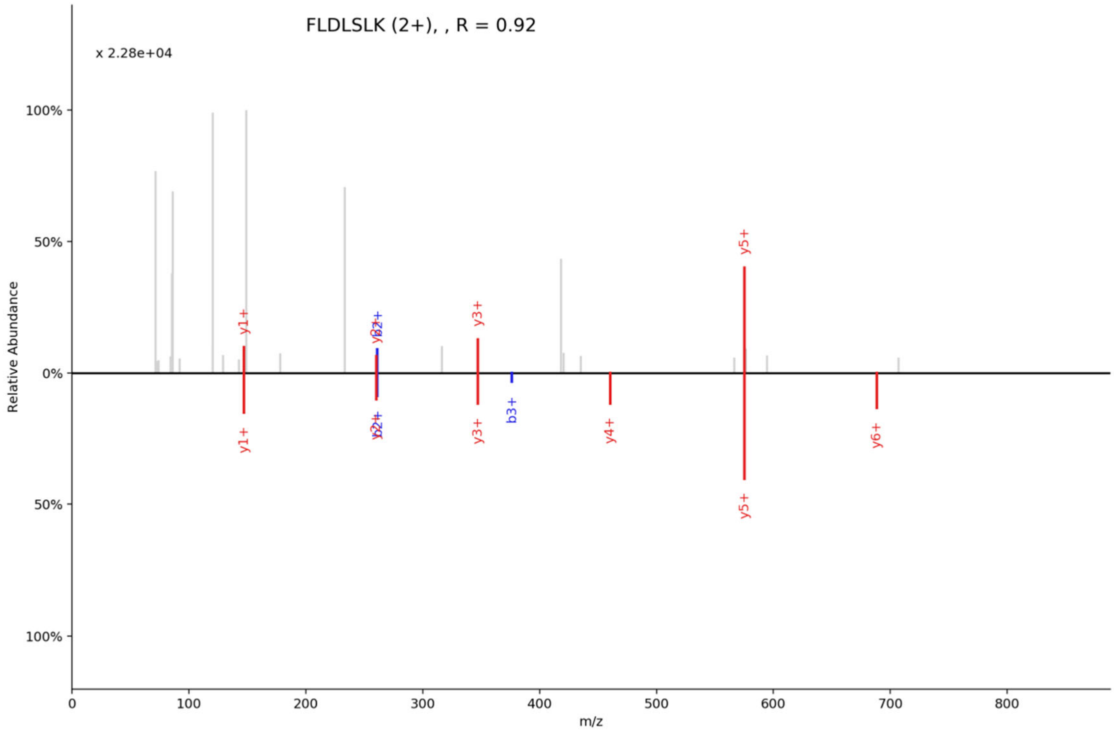

Figure S19. Mass spectrum of peptide P18

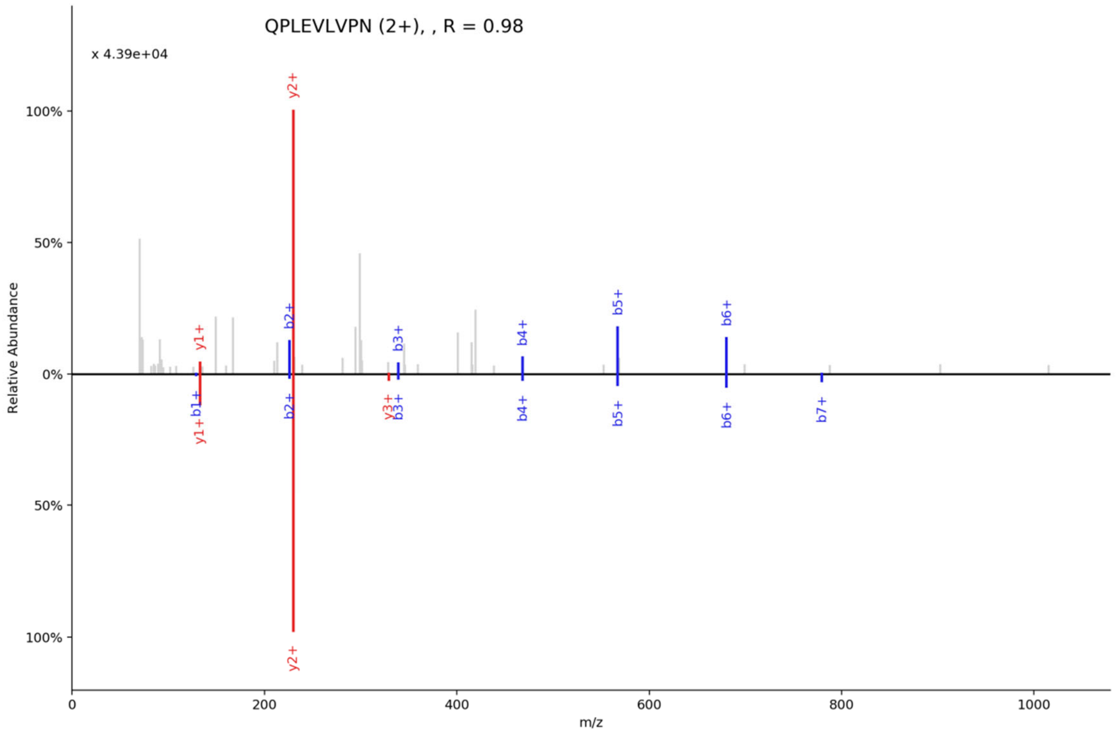

Figure S20. Mass spectrum of peptide P19

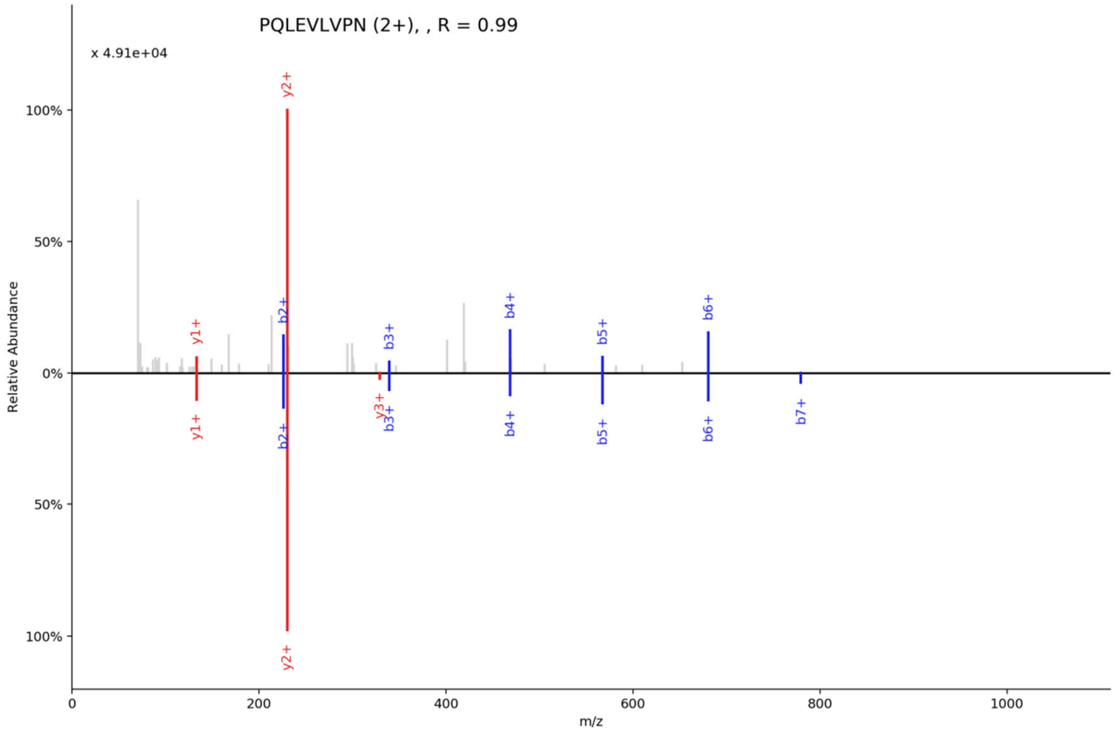

Figure S21. Mass spectrum of peptide P20

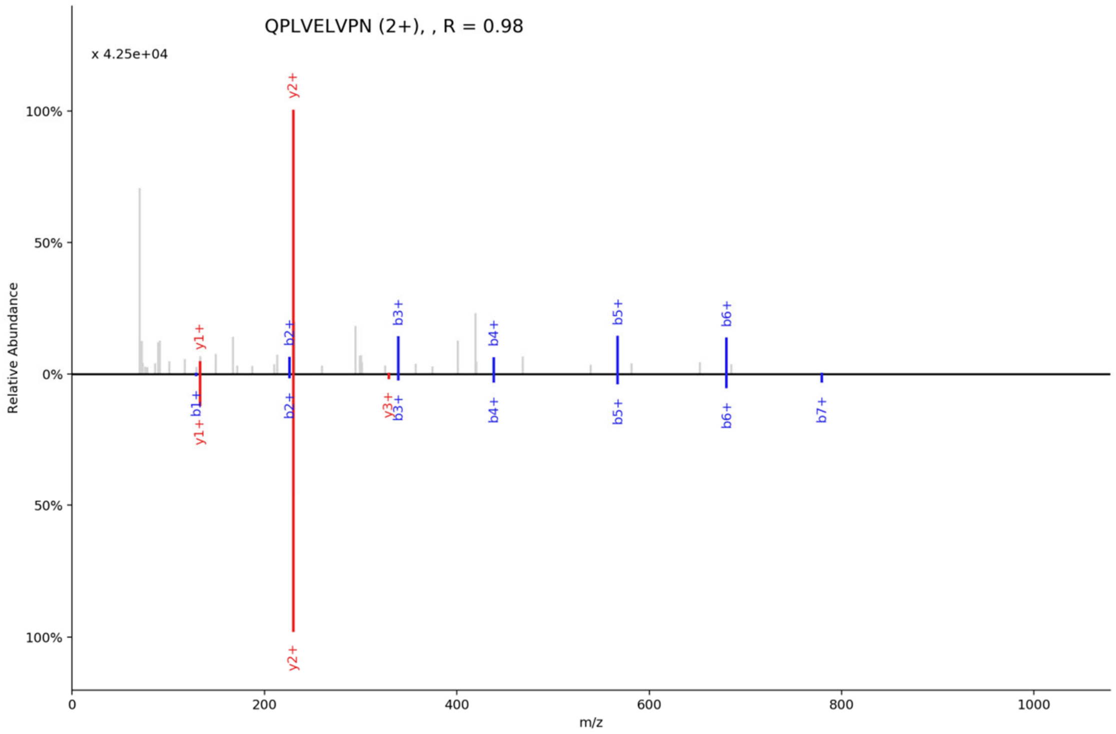

Figure S22. Mass spectrum of peptide P21

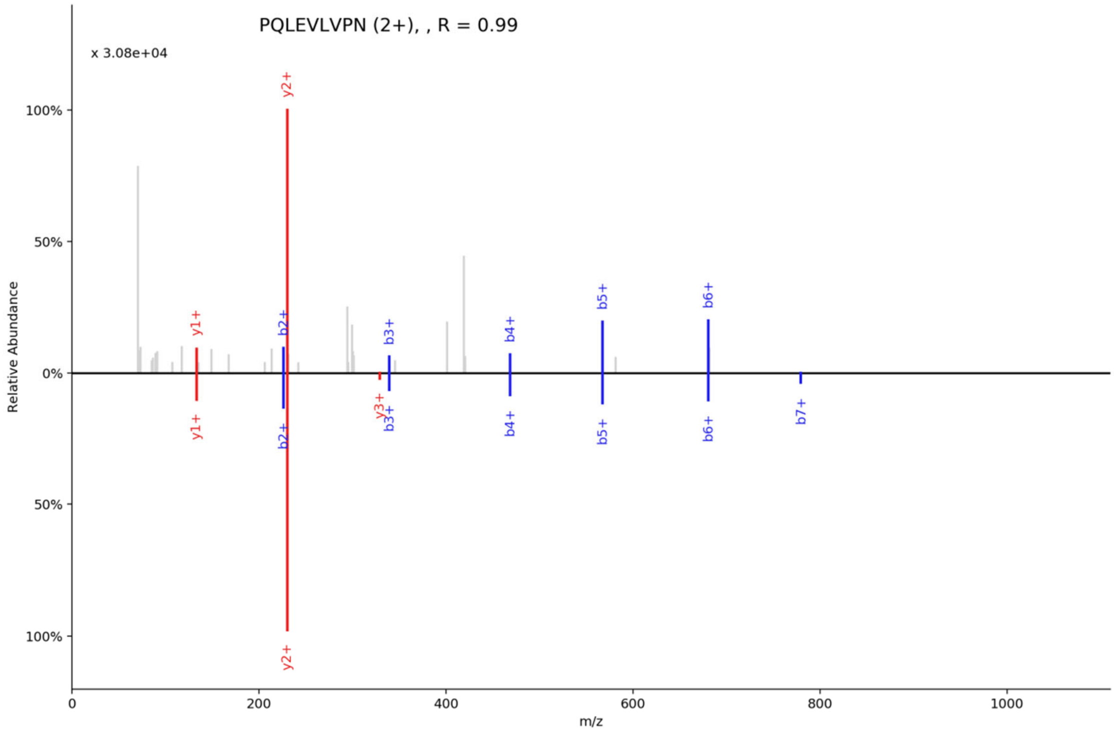

Figure S23. Mass spectrum of peptide P22

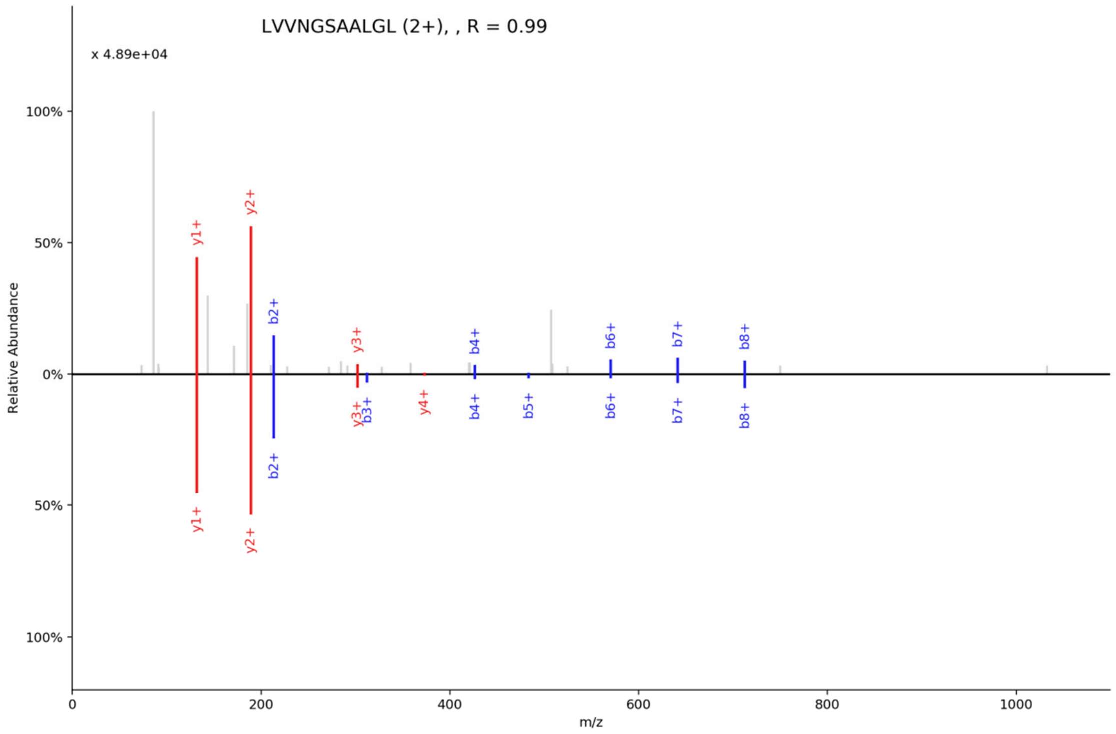

Figure S24. Mass spectrum of peptide P23

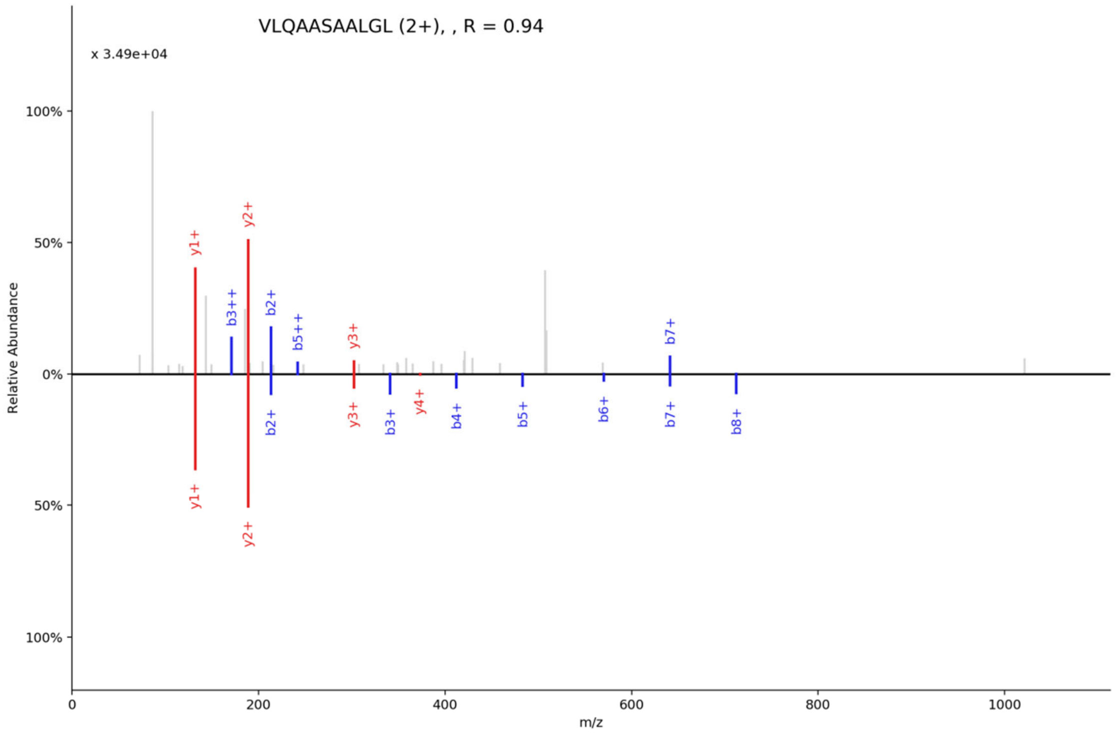

Figure S25. Mass spectrum of peptide P24

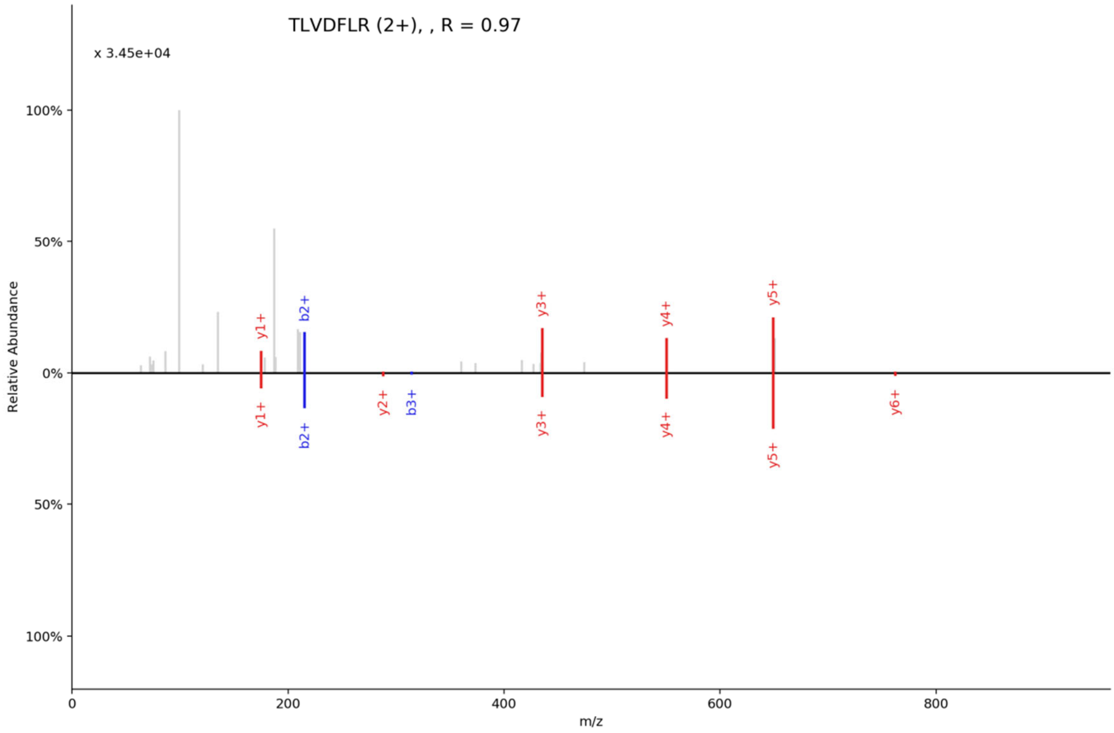

Figure S26. Mass spectrum of peptide P25

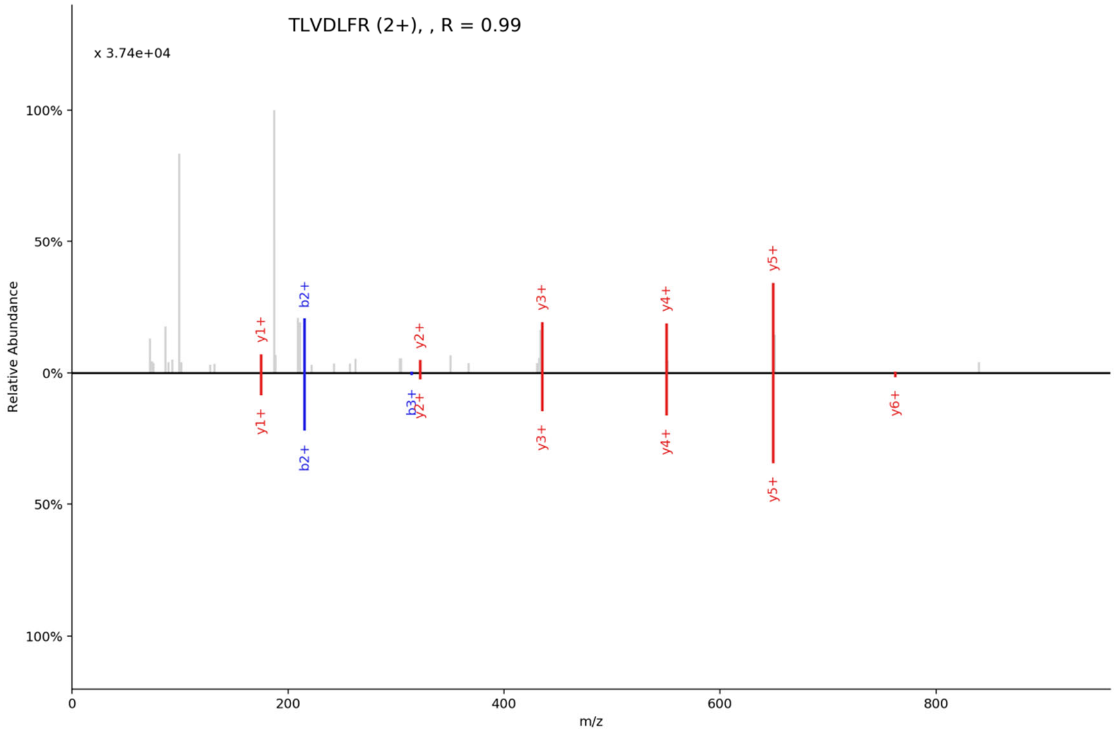

Figure S27. Mass spectrum of peptide P26

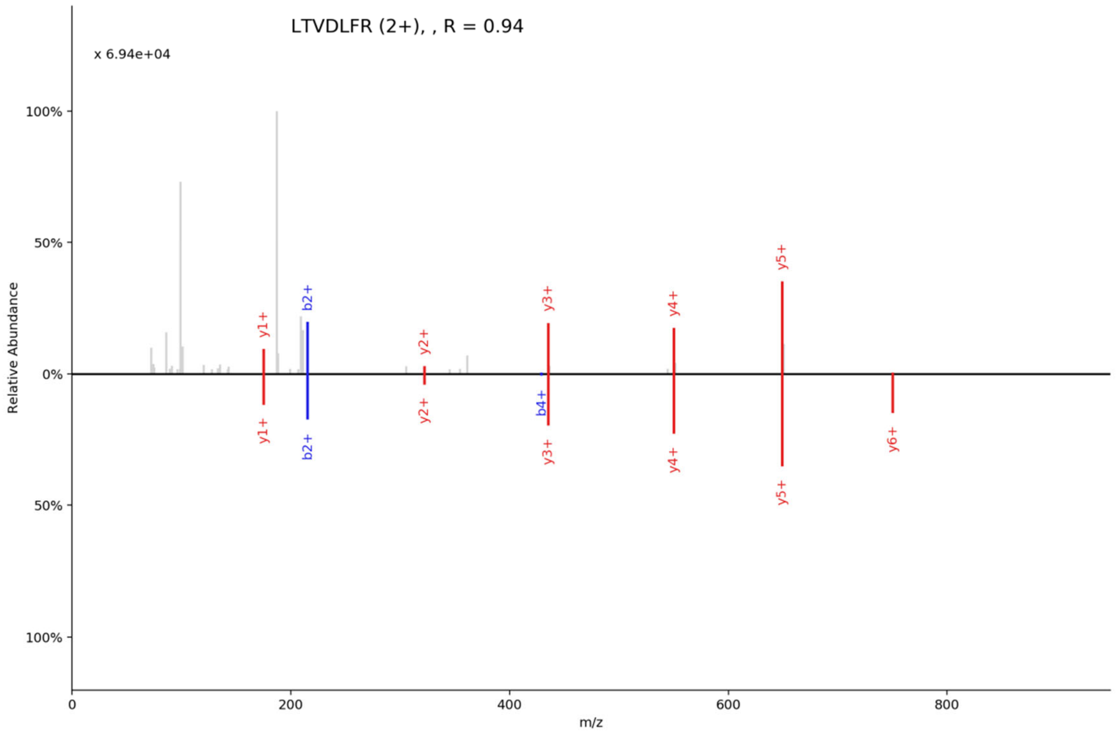

Figure S28. Mass spectrum of peptide P27

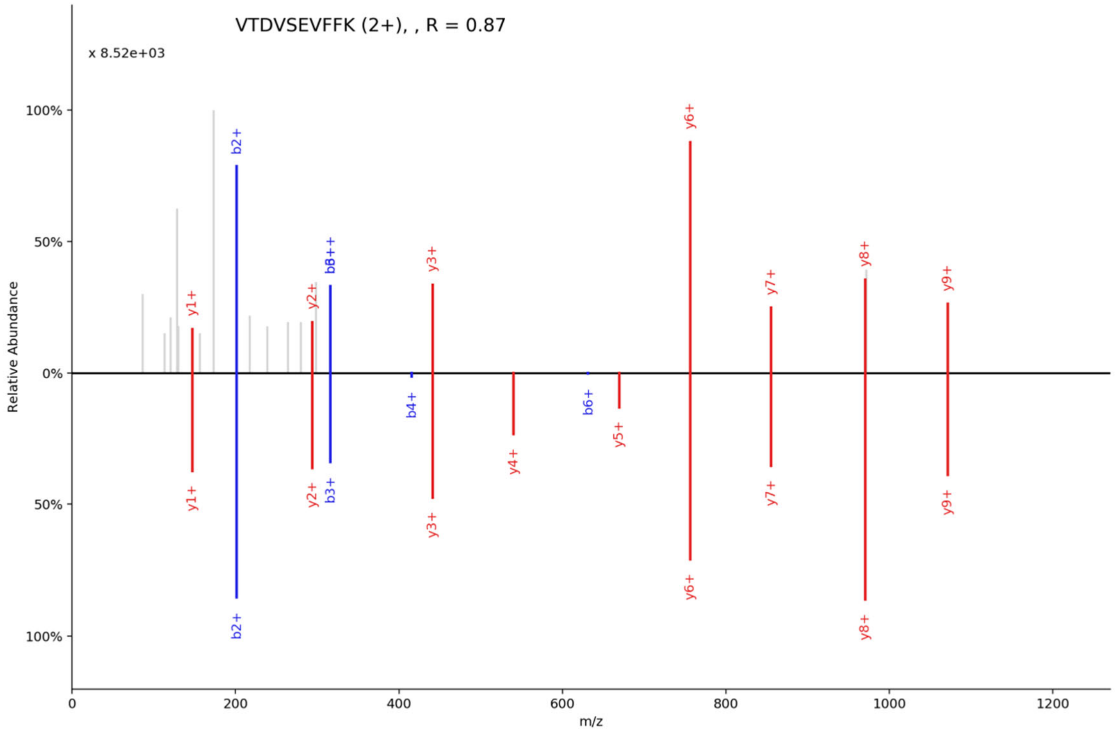

Figure S29. Mass spectrum of peptide P28

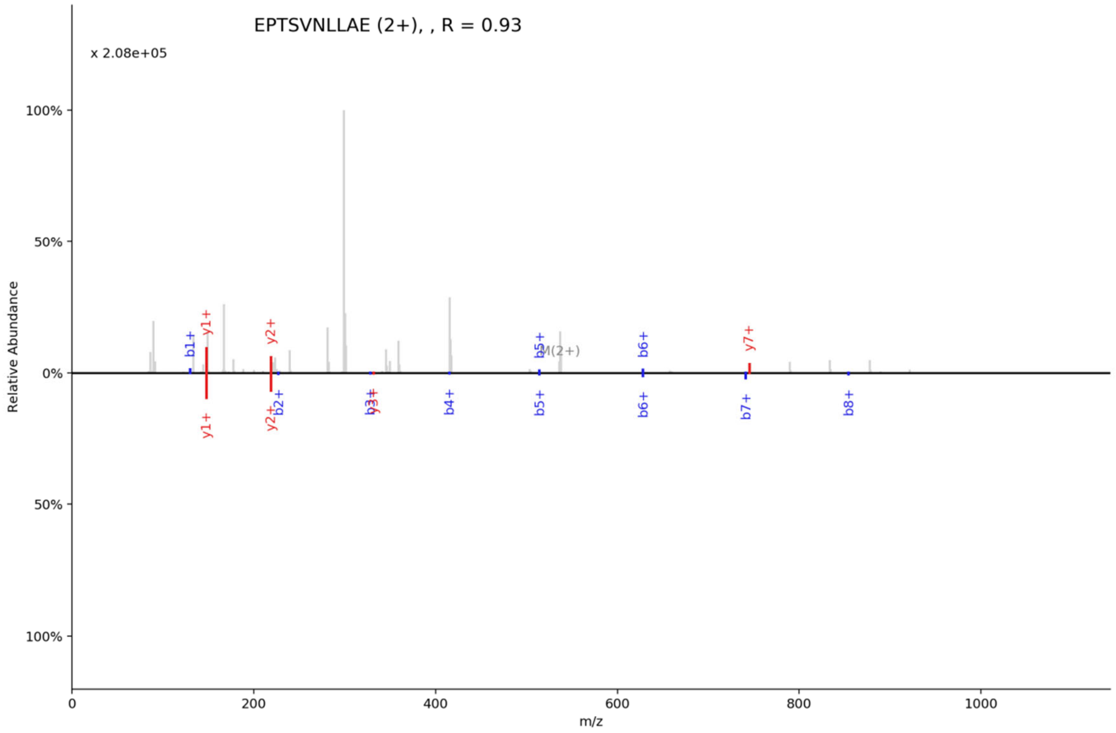

Figure S30. Mass spectrum of peptide P29

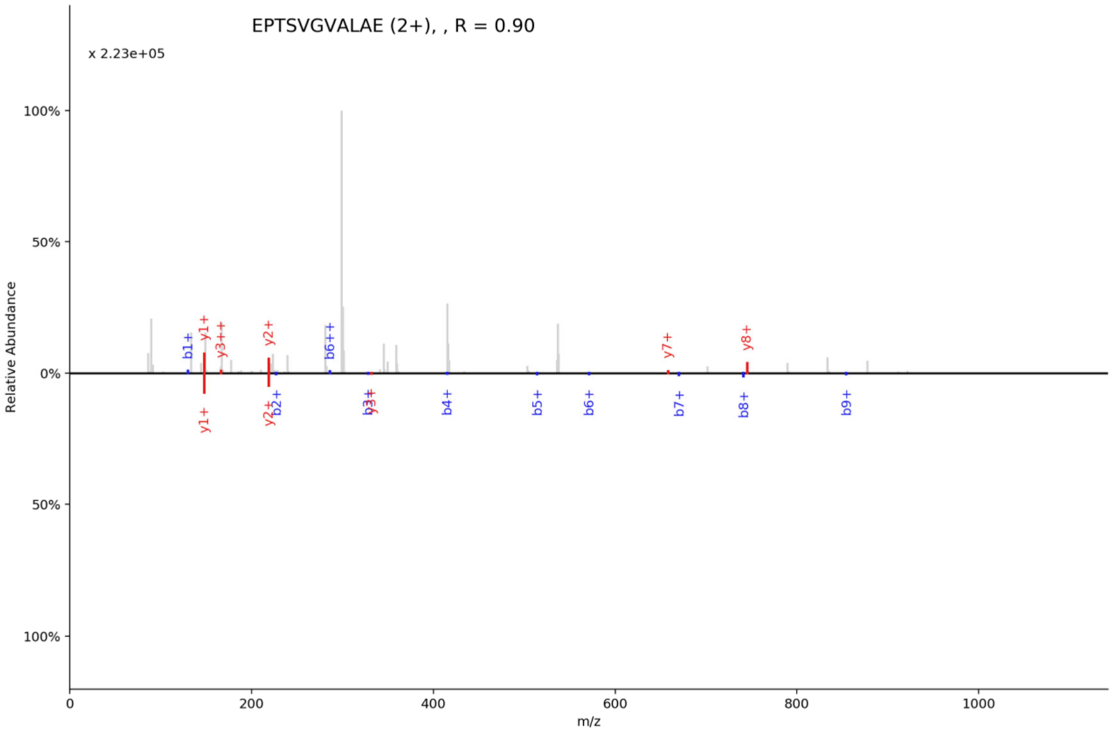

Figure S31. Mass spectrum of peptide P30
