# Supplementary material for: Identification of Bioactive Peptides from Caenorhabditis elegans Secretions That Promote Indole-3-Acetic Acid Production in Arthrobacter pascens ZZ21
Source: Microorganisms. 2025 Aug 21;13(8):1951. doi: 10.3390/microorganisms13081951 (PMC12388801; doi:10.3390/microorganisms13081951)
Supplement: Supplementary file 1 [file microorganisms-13-01951-s001.zip › Supplementary Tables.pdf]

Table S1. Solubility assessment of 30 candidate peptides

No.	Purity (%)	Solubilty in ultrapure water	Solubilty in DMSO	Solubilty in 0.1 M PBS
P1	99.7	≤5 mg/mL	≤10 mg/mL	N/A[a]
P2	98.0	≤10 mg/mL	≤10 mg/mL	≤5 mg/mL
P3	98.4	≤10 mg/mL	≤10 mg/mL	≤10 mg/mL
P4	99.5	≤10 mg/mL	≤10 mg/mL	≤5 mg/mL
P5	97.4	≤5 mg/mL	≤10 mg/mL	N/A
P6	96.9	≤5 mg/mL	≤10 mg/mL	≤5 mg/mL
P7	99.5	≤5 mg/mL	≤10 mg/mL	≤5 mg/mL
P8	99.3	≤5 mg/mL	≤10 mg/mL	≤5 mg/mL
P9	95.1	≤5 mg/mL	≤10 mg/mL	≤5 mg/mL
P10	96.5	≤10 mg/mL	≤10 mg/mL	≤5 mg/mL
P11	99.7	≤10 mg/mL	N/A	N/A
P12	95.3	N/A	≤10 mg/mL	≤5 mg/mL
P13	97.9	N/A	≤10 mg/mL	N/A
P14	98.6	N/A	≤10 mg/mL	N/A
P15	96.6	≤5 mg/mL	≤10 mg/mL	≤5 mg/mL
P16	98.8	≤5 mg/mL	≤10 mg/mL	≤5 mg/mL
P17	95.4	≤10 mg/mL	≤10 mg/mL	≤10 mg/mL
P18	96.7	N/A	≤10 mg/mL	N/A
P19	95.7	≤5 mg/mL	≤10 mg/mL	≤5 mg/mL
P20	96.1	N/A	≤10 mg/mL	N/A
P21	95.6	≤5 mg/mL	≤10 mg/mL	≤5 mg/mL
P22	96.6	N/A	≤10 mg/mL	N/A
P23	96.5	≤5 mg/mL	≤10 mg/mL	≤5 mg/mL
P24	97.2	N/A	≤10 mg/mL	N/A
P25	99.1	N/A	≤10 mg/mL	N/A
P26	99.5	≤10 mg/mL	≤10 mg/mL	≤5 mg/mL
P27	99.6	N/A	≤10 mg/mL	N/A
P28	99.3	N/A	≤15 mg/mL	N/A
P29	96.7	N/A	≤10 mg/mL	N/A
P30	96.3	N/A	N/A	≤5 mg/mL

[a]: N/A indicates solubility lower than 0.1 mg/mL.

Table S2. The standard curves of P1, P9, P19, P25 and P26

Peptides Sequences	Q2b	linear equations	R^2 value	Dynamic Concentration Range (fmol)	LOQ (fmol)
GNALATR	y5	y=14709.98x-3270798.18	0.9985	200-2000	200
	y1	y=7538.90x-1450650.08	0.9994	200-2000	200
	b2	y=14703.44x-3255433.82	0.9986	200-2000	200
QHGLPQEV	b3	y=19142.00x-3930275.18	0.9745	200-2000	200
	b4	y=38112.26x-5459343.83	0.9302	200-2000	200
	b5	y=12683.07x-2529030.20	0.9597	200-2000	200
QPLEVLVPN	y3	y=9942.76x-145582.97	0.9691	100-1000	100
	y2	y=342760.48x+14185421.63	0.9655	100-1000	100
	b5	y=36777.03x+3348773.33	0.9285	100-1000	100
TLVDFLR	y5	y=219456.37x-24861390.30	0.9504	50-1250	50
	y3	y=103237.69x-11266208.16	0.9541	50-1250	50
	y1	y=34280.38x-2071656.68	0.9360	50-1250	50
TLVDLFR	y5	y=249630.35x+12007799.31	0.9921	20-1000	20
	y4	y=108245.99x+5647647.30	0.9904	20-1000	20
	y3	y=120386.35x+7578972.06	0.9916	20-1000	20
